# Meta-Analysis of Dietary Tannins in Small Ruminant Diets: Effects on Growth Performance, Serum Metabolites, Antioxidant Status, Ruminal Fermentation, Meat Quality, and Fatty Acid Profile

**DOI:** 10.3390/ani15040596

**Published:** 2025-02-19

**Authors:** Asma Al Rharad, Soufiane El Aayadi, Claire Avril, Alassane Souradjou, Fafa Sow, Younouss Camara, Jean-Luc Hornick, Soumaya Boukrouh

**Affiliations:** 1 Laboratory of Physical-Chemistry of Materials, Natural Substances and Environment, Department of Chemistry, Faculty of Science and Technology, Abdelmalek Essâadi University, Tangier 90000, Morocco; 2 Animal Production Department, Institut Agronomique et Veterinaire Hassan II, Medinat Al Irfane, Rabat 10112, Morocco; 3 AgroBiosciences et Chimie, Haute École Provinciale de Hainaut Condorcet, 7800 Ath, Belgium; 4 Department of Veterinary Management of Animal Resources, Faculty of Veterinary Medicine, University of Liège, 4000 Liège, Belgium; 5 Senegalese Institute for Agricultural Research (ISRA), Dakar PB 3120, Senegal; 6 Département Productions Animales et Élevage (PAE), UFR Sciences Agronomiques, Aquaculture et Transformation Agroalimentaire (S2ATA), Université Gaston Berger, Saint-Louis BP 234, Senegal

**Keywords:** tannins, sheep, goat, intake, digestibility, antioxidant, meat quality, fatty acid profile

## Abstract

Tannins are secondary metabolites naturally present in various plants as a defense mechanism. In animal nutrition, they play a dual role, acting as both antinutritional factors and bioactive agents, with potential benefits in nutrient metabolism and product quality. This meta-analysis aimed to evaluate the effects of dietary tannin on nutrient intake and digestibility, blood serum metabolite levels, growth performance, carcass characteristics, and meat quality in small ruminants. Ninety-seven studies were included in this meta-analysis. The effects of tannins were analyzed using a random-effects model to determine the weighted mean difference between treatments with tannins and the control (without tannins). Publication bias and heterogeneity between studies were explored, and meta-regression and subgroup analyses were performed. The results indicated that tannin significantly increased the intake of dry matter, crude protein, nitrogen (N), fecal N, and meat fatty acids (FA), such as C18:2 ω6, C18:3 ω3, C20:4 ω6, C20:5 ω3, total ω3 and ω6 FA, and total polyunsaturated FA (PUFA). However, NH_3_-N, urinary N, blood urea nitrogen, cold carcass weight, subcutaneous fat thickness, drip loss, and nutrient digestibility decreased. In conclusion, tannin in small ruminants did not affect animal productivity and antioxidative status, but mainly modified nitrogen metabolism and improved the fatty acid profile of meat.

## Simple Summary

Tannins are plant-derived secondary metabolites with both antinutritional and bioactive properties, influencing nutrient metabolism, animals’ performance, and product quality. This meta-analysis of 97 studies assessed the effects of dietary tannin in small ruminants and showed improvements in nutrient intake, nitrogen metabolism, and meat fatty acid profile, including omega-6 and omega-3 fatty acids. However, tannins affected nutrient digestibility, ammonia nitrogen levels, and certain carcass characteristics. Although tannins did not significantly affect productivity or antioxidative status, they proved effective in modulating nitrogen utilization and enhancing meat quality.

## 1. Introduction

Small ruminants are major contributors to the income of people in rural areas and are managed in rangelands, primarily under extensive and semi-extensive grazing systems [1]. However, rangelands face several challenges, including land degradation, reduced biodiversity, and water scarcity, which negatively affect animal productivity and the environment [2,3]. This has raised the need to exploit alternative feed resources that are both cost-effective and environmentally sustainable [4,5,6]. These alternative feed resources include agricultural and industrial byproducts and unconventional plant sources that can supply essential nutrients without straining existing resources. Generally, these alternative feed resources are highly concentrated in plant secondary compounds, such as tannins [7]. Tannins are a diverse group of polyphenolic compounds that are mainly classified as condensed (CT) and hydrolyzable (HT) according to their chemical structure. CTs are oligomers and polymers of connected flavan-3-ol units, whereas HTs are polyol cores attached to gallic acid or ellagic acid esters [8]. Tannins are reported to be environmentally friendly, as they minimize the ruminal production of enteric methane (CH_4_) while improving the growth rate and feed conversion efficiency [9]. Moreover, nitrous oxide (N_2_O) emissions from livestock systems can be manipulated using tannin-rich plants because of their ability to form complexes with proteins and carbohydrates in the rumen [10].

In the meat industry, the economic value of animals is primarily defined by carcass weight, dressing percentage, and fat [11]. Tannins present in small ruminant diets have been linked to alterations in these parameters, particularly their effects on feed intake and digestibility, growth performance, and meat quality [12,13,14,15,16]. Additionally, tannins can interact with muscle proteins, thereby affecting the water-holding capacity, which is a critical determinant of meat juiciness and tenderness [17]. Color is also important because it affects sensory acceptability and visual appeal [17]. Several studies have demonstrated the positive effects of tannins on carcass characteristics and meat quality. However, these results are inconsistent and contradictory. Owing to their antioxidant properties, tannins have been reported to either prevent lipid oxidation and improve color stability in meat or have no impact, depending on their inclusion level [16]. Moreover, tannins have been reported to either inhibit FA biohydrogenation or enhance Δ9-desaturase activity in ruminant muscles [18]. This activity consistently leads to an increase in conjugated linoleic acid (CLA), including trans-11 18:1 (VA: vaccenic acid), and the preservation of dietary polyunsaturated FA (PUFA) [18]. These changes have been linked to a reduced incidence of diabetes and cancer and improved cardiovascular health among consumers [18]. Variations in carcass and meat fatty acid profiles when tannins are included in ruminant diets vary according to the literature and may be attributed to factors such as animal age and species, type and level of tannins administered, feeding duration, the composition of the basic diet, and overall management of the production system [18].

Small ruminants, especially goats, are considered more tolerant to tannins than larger ruminants, probably because of their evolutionary adaptation to browsing trees and shrubs, which often contain high levels of tannins [13,15,19]. Some authors have suggested that the exposure of goats to CT enhances the secretion of proteins in the parotid saliva. Parotid salivary protein levels vary according to tannin concentration and animal species [20]. It was significantly lower in goats (212 μg/mL) fed no CT wheat-straw-containing diet than in goats (550 μg/mL) fed *Ceratonia silique* higher CT-containing diet. Moreover, compared to cattle and sheep, goat parotid saliva is rich in glycine (6.1%), proline (6.5%), and glutamine (16.5%), which are known to enhance the affinity of proteins for CT because of the formation of stable complexes within the pH range of their digestive apparatus [20]. Tannin-proline-rich protein complexes reduce the negative influence of protein-tannin binding on feed taste and intake. They may also mitigate the detrimental effects of tannins on ruminal microbiota and their enzymatic activity [21]. *Streptococcus caprinus*, isolated from goats grazing tannin-rich Acacia species, has been shown to degrade CT, which testifies to the ability of goats to consume high CT-containing feed [22].

Meta-analysis is a statistical tool that analyzes a combination of quantitative data from various studies and determines the factors that influence the results, thus improving the generalizability of conclusions [23]. Therefore, interest in the application of MA in animal nutrition is increasing. Meta-analysis studies have already been published on the effects of tannins on cattle as large ruminants; however, no meta-analysis has addressed the impact of tannins, especially on goats. Moreover, there is a need to understand the effects of animals’ initial age and weight, experimental duration, tannin concentration, type and administration form, the level of concentrate in the diet, and most importantly, the small ruminant species (goat and sheep) on the response to dietary tannins.

We hypothesized that tannin would enhance the productivity and nutritional value of small ruminant meat and its potential health benefits. The objectives of this meta-analysis were to assess the effects of tannins on small ruminant nutrient intake and digestibility, blood parameters, growth performance, carcass characteristics, and meat quality, and to analyze the factors that affect the response of small ruminants to tannin.

## 2. Materials and Methods

### 2.1. Literature Search and Study Selection

**Figure 1:**
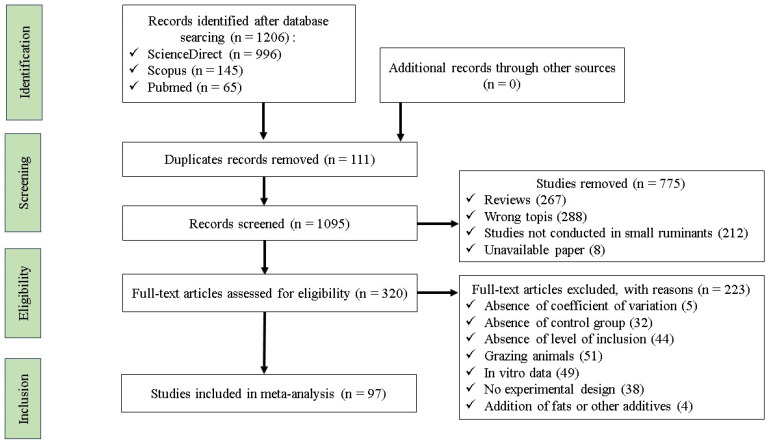
PRISMA flow chart explaining the literature search strategy and study selection for the meta-analysis.

A comprehensive search of articles published in English was conducted to identify studies that examined the effects of tannin-containing feed in the diet of small ruminants on feed intake, nutrient digestibility and fermentation, blood parameters, carcass characteristics, meat nutritional quality, and fatty acid (FA) profile. The process of identifying, screening, assessing eligibility, and including studies followed the Preferred Reporting Items for Systematic Reviews and Meta-Analyses (PRISMA) standards (Figure 1) [24]. The keywords “tannins”, AND “goat OR sheep OR lamb”, AND “meat” were used to search for relevant papers in Scopus, PubMed, Google Scholar, and ScienceDirect. A total of 97 articles were identified (Supplementary Table S1). Duplicate papers from more than one database were removed using Zotero software (version 6.0.3), and only papers published between 2000 and 2023 were retained. First, the identified papers were screened based on their abstracts and titles. We excluded reviews and meta-analyses, publications concerning animals other than small ruminant species, studies that examined infected animals, in vitro experiments, and studies that did not report any variables of interest.

Second, for inclusion in the final database, the articles analyzed had to satisfy a set of the following pre-established inclusion criteria: (1) original articles published in English; (2) in vivo experiments evaluating the effects of tannin in small ruminants (goats and sheep); (3) studies that did not incorporate additional dietary additives that could complicate the interpretation of the results; (4) studies detailing the concentration of tannins or adequate data to estimate the amount of tannins included in the diets; (5) data available on carcass characteristics and meat quality; and (6) adequate information on the research methodology, including animal randomization, number of animals involved, and measures of variance, such as standard deviation or standard error.

### 2.2. Data Extraction

After applying the inclusion criteria, the final database comprised 97 peer-reviewed articles. Data were extracted for variables reported in at least three separate studies, including the mean, number of repetitions, and standard deviation (SD) in the experimental and control groups. When the SD was not reported, the SD value was calculated by multiplying the standard error of the mean (SEM) by the square root of the number of animals in each group. When a single study utilized different incorporation levels, each level was considered a treatment and compared to the same control group.

The following variables were included in the final database: intake of dry matter (DMI), forage (DMI-F), crude protein (CPI), neutral detergent fiber (NDFI), digestibility of DM (DMD), CP (CPD), NDF (NDFD), acid detergent fiber (ADFD), hemicellulose (HCellD), and cellulose (CellD). The fermentation variables were ruminal pH, NH_3_-N, and short-chain fatty acids (SCFA)—such as butyrate, propionate, acetate—total volatile fatty acids (TVFA), and the acetate: propionate ratio. The variables for nitrogen metabolism included N intake, urine N, fecal N, and retained nitrogen. Blood metabolites included hemoglobin, glucose, total protein, globulin, creatinine, albumin, and blood urea nitrogen (BUN), and enzymes such as alkaline phosphatase (ALP), alanine transaminase (ALT), and aspartate aminotransferase (AST),

For carcass and meat quality parameters, data were extracted for response variables, including final body weight (FBW), average daily gain (ADG), feed conversion ratio (FCR), cold and hot carcass weights (CCW and HCW); meat physicochemical parameters such as Warner-Bratzler shear force (WBSF), cooking loss, drip loss, moisture, proteins, ash, and fat; and color parameters such as redness (a*), lightness (L*), and yellowness (b*). The fatty acid profiles included stearic (C18:0), oleic (C18:1 ω9), vaccenic (C18:1 t11), linoleic (LA, C18:2 ω6), conjugated linoleic (CLA, C18:2 c9t11), α-linolenic (ALA, C18:3 ω3), arachidonic (ARA, C20:4 ω6), eicosapentaenoic (EPA, C20:5 ω3), docosapentaenoic (DPA, C22:5 ω3), docosahexaenoic (DHA, C22:6 ω3), total ω3 and ω6 FA, ω6:ω3, total saturated FA (SFA), total monounsaturated FA (MUFA), total polyunsaturated FA (PUFA), ∆9C16, and ∆9C18.

Additional information was extracted from the included papers regarding the following: (1) authors and publication year, (2) tannin concentration in the diet (g/kg DM), (3) tannin type (condensed, hydrolyzable, or total tannins), (4) type of administration (extract or present in plants), (5) the initial weight of animals, (6) their age, (7) the experimental duration, (8) the country in which the study was conducted, and (9) the amount of concentrate in the diet.

### 2.3. Statistical Analysis

Statistical analysis of the database was performed using a meta-analysis approach with R software (version 4.4.1, R Core Team, Vienna, Austria). The effect of incorporating tannin-containing feed into the diets of small ruminants was assessed by calculating the weighted mean differences (WMD) between the diets containing tannins and control (diets without tannins) groups with a 95% confidence interval (CI). Weighing was performed by multiplying the treatment means by the inverse of their variance, employing the random-effects model method suggested by DerSimonian and Laird [25].

The Chi-square (Q) test and Inconsistency index (I^2^) statistics were used to evaluate the heterogeneity of the treatment effect, with heterogeneity assumed to exist when the probability was less than 0.05 [26]. The I^2^ statistic measures the proportion of variation between studies attributed to heterogeneity rather than chance, with values ranging from 0% to 100%. An I^2^ value < 25% shows low heterogeneity, I^2^ values ranging between 25% and 50% stipulate moderate heterogeneity, and I^2^ values > 50% signify high heterogeneity [27].

Publication bias was assessed using Egger’s regression asymmetry and Begg’s rank correlation tests [28,29] for all outcomes, with bias considered present when *p* ≤ 0.05. In cases where these two parameters were significant, Rosenberg’s fail-safe number (NF) was applied as a third test. NFs are generally deemed robust when they exceed 5 × Ne + 10, where Ne represents the number of papers in each treatment group [30].

Meta-regression was used to determine sources of heterogeneity if the following criteria were met: (1) the presence of high heterogeneity (I^2^ statistic > 50% or *p* ≤ 0.10); (2) the absence of publication bias [28,29,30]; and (3) response variables present in at least 10 separate studies [31]. All meta-regression analyses employed the DerSimonian and Laird method of moments, an approach designed to estimate between-study variance. A mixed model was used to adjust the data, with the WMD serving as the dependent variable. The mixed-effect model was defined as follows:

ϴi = β + βi xij + … + βiq xiq + µi

where ϴi = the true effect treatment in the ith explanatory variable; β = the overall true effect treatment; xij = the value of the jth variable (j = 1, 2, …, q) for the ith explanatory variable; βi = change in the true size effect for a unit increase in the jth variable; and μi∼N (0 t^2^). The t^2^ indicates the heterogeneity that is not accounted for by the variable.

Subgroup analysis was conducted to evaluate the WMD for covariates that showed significance with a *p*-value ≤ 0.05. In the meta-regression analysis, WMD was evaluated using subgroup analysis for covariates with *p* ≤ 0.05. The proportion of between-study variance explained by the variables was determined using the adjusted R^2^. The latter variable was calculated by comparing the estimated between-study variance with the variables included in the model (σ^2^) and the variance when the variables were excluded (σo^2^).

Adjusted R^2^ = (σo^2^ − σ^2^)/σo^2^σo

Dietary tannin concentration (0–20, 21–40, 41–100, >100 g/kg DM), animal age (1–6, 7–12, 13–24, 25–48 months), initial weight (7–20, 21–40, 41–60, 61–71 kg), quantity of concentrate present in the diets (<400, 400–600, >600 g/kg DM), and feeding duration (5–30, 31–90, 91–195 days) were considered continuous covariates, whereas the continent in which the study was conducted (North and South America, Europe, Asia, and Africa), type of tannins (HT, CT, TT), and tannin distribution form (extract or naturally present in the diet) were considered categorical covariates.

## 3. Results

This meta-analysis investigated the variable impact of tannins on small ruminant performance, particularly focusing on their effects on carcass characteristics and meat quality, which are substantial indicators of the economic value of livestock. The dataset comprised 97 peer-reviewed publications with 3143 treatment means. Tables 1–4 delineate the results of the combined effect size, heterogeneity, and publication bias of the treatments.

### 3.1. Intake, Digestibility of Nutrients, and Fermentation Parameters

The results revealed that tannin in small ruminant diets significantly improved DMI and DMI-F (*p* < 0.001) (Table 1), whereas no significant effect was observed on CPI (*p* = 0.169) or NDFI (*p* = 0.266). Tannins in small ruminant diets reduced all digestibility parameters, including DMD, CPD, NDFD, and ADFD (*p* < 0.01), whereas no significant effects were observed for HCellD (*p* = 0.181) or CellD (*p* = 0.576).

Concerning fermentation parameters, tannins reduced NH_3_-N (*p* < 0.001) and increased butyrate (*p* = 0.039). No significant effects were observed for ruminal pH (*p* = 0.123), acetate (*p* = 0.526), propionate (*p* = 0.261), total volatile FA (*p* = 0.537), or the acetate: propionate ratio (*p* = 0.152).

**Table 1:** Effects of dietary tannin on feed intake, nutrient digestibility, and fermentation parameters of small ruminants.

	NS	NC	Tannins		Heterogeneity		Bias (*p*-Value)			NFs
			WMD (95% CI)	*p*-Value	I^2^ (%)	*p*-Value	Egger	Begg	NFs	
Intake (g/day)										
Dry matter (DMI)	66	175	0.65 (0.36, 0.95)	<0.001	88.67	<0.001	<0.001	0.001	<0.001	7314
Forage (DMI-F)	10	25	0.92 (0.38, 1.46)	<0.001	78.52	<0.001	<0.001	<0.001	<0.001	376
Crude protein	20	47	0.27 (−0.12, 0.65)	0.169	75.96	<0.001	<0.001	0.020	<0.001	155
NDF	23	53	0.16 (−0.12, 0.45)	0.266	63.26	<0.001	<0.001	0.084	0.002	118
Digestibility (g/kg DM)										
Dry matter	38	79	−0.57 (−0.87, −0.27)	<0.001	76.16	<0.001	<0.001	<0.001	<0.001	1285
Crude protein	37	79	−0.56 (−0.91, −0.21)	0.002	81.51	<0.001	0.234	0.092	<0.001	982
NDF	41	88	−0.75 (−1.11, −0.38)	<0.001	84.76	<0.001	0.001	0.001	<0.001	2379
ADF	28	57	−0.84 (−1.30, −0.37)	<0.001	85.10	<0.001	0.994	0.022	<0.001	1124
Hemicellulose	6	20	0.25 (−0.11, 0.61)	0.181	40.66	0.018	0.806	0.153	0.040	3
Cellulose	4	10	0.16 (−0.41, 0.74)	0.576	47.35	0.039	0.004	0.037	0.186	0
Fermentation parameters										
Ruminal pH	24	52	0.20 (−0.05, 0.45)	0.123	53.22	<0.001	0.772	0.152	0.015	40
NH_3_-N (mg/dL)	25	54	−0.43 (−0.67, −0.18)	<0.001	52.10	<0.001	0.031	0.006	<0.001	437
SCFA (mol/100 mol)										
Acetate	17	36	−0.10 (−0.40, 0.20)	0.526	56.60	<0.001	<0.001	<0.001	0.160	0
Propionate	17	36	0.25 (−0.19, 0.68)	0.261	77.87	<0.001	<0.001	<0.001	0.064	0
Butyrate	16	34	0.29 (0.01, 0.56)	0.039	46.44	0.001	0.875	0.791	0.003	65
Acetate:propionate	16	35	−0.32 (−0.77, 0.12)	0.152	79.06	<0.001	<0.001	<0.001	0.010	35
TVFA	16	33	0.11 (−0.23, 0.44)	0.537	66.73	<0.001	0.927	0.549	0.156	0

NS: number of studies; NC: number of comparisons between tannins and control treatment; WMD: weighted mean differences between treatments with tannins and control; CI: confidence interval of WMD; *p*-value to χ2 (Q) test of heterogeneity; I^2^: proportion of total variation of size effect estimates that is due to heterogeneity; Egger’s regression asymmetry test; Begg’s rank correlation method; NFs: Rosenberg’s fail-safe number; DMI: dry matter intake; DMI-F: dry matter intake of forage; NDF: neutral detergent fiber; ADF: acid detergent fiber; NH_3_-N: nitrogen ammonia; SCFA: short-chain fatty acids; TVFA: total volatile fatty acids.

### 3.2. Nitrogen Metabolism and Blood Parameters

Nitrogen metabolism was significantly affected by tannin incorporation in small ruminant diets (Table 2). Dietary tannin increased N intake (*p* = 0.002), urinary N (*p* < 0.001), and fecal N (*p* = 0.002), but had no significant effect on retained N (*p* = 0.146).

BUN levels were significantly reduced by tannin incorporation (*p* < 0.001), whereas no significant effects were observed for hemoglobin (*p* = 0.152), glucose (*p* = 0.285), total protein (*p* = 0.065), globulin (*p* = 0.347), albumin (*p* = 0.459), ALT (*p* = 0.120), AST (*p* = 0.845), ALP (*p* = 0.598), or creatinine (*p* = 0.727).

**Table 2:** Effects of dietary tannin on feed nitrogen metabolism and blood parameters of small ruminants.

	NS	NC	Tannins		Heterogeneity		Bias (*p*-Value)			NFs
			WMD (95% CI)	*p*-Value	I^2^ (%)	*p*-Value	Egger	Begg	NFs	
Nitrogen metabolism (g/day)										
N intake	30	62	0.52 (0.19, 0.85)	0.002	74.74	<0.001	<0.001	0.057	<0.001	924
N urine	29	59	−0.70 (−1.17, −0.23)	<0.001	85.9	<0.001	<0.001	<0.001	<0.001	796
N fecal	28	53	0.74 (0.26, 1.21)	0.002	84.52	<0.001	0.002	0.008	<0.001	846
N retained	24	49	0.38 (−0.13, 0.89)	0.146	86.38	<0.001	<0.001	0.202	<0.001	237
Blood parameters										
Hemoglobin (g/dL)	10	29	0.16 (−0.06, 0.37)	0.152	0	0.73	0.011	0.002	0.045	2
Total protein (g/dL)	19	51	0.18 (−0.01, 0.38)	0.065	34.12	0.004	0.448	0.909	0.013	44
Globulin (g/dL)	3	11	0.16 (−0.17, 0.48)	0.347	0	0.755	0.257	0.087	0.135	0
Albumin (g/dL)	16	44	0.09 (−0.14, 0.31)	0.459	42.75	0.001	0.35	0.279	0.148	0
ALT (U/L)	14	38	−0.18 (−0.46, 0.10)	0.12	53.16	<0.001	0.05	0.126	0.029	13
AST (U/L)	13	38	−0.02 (−0.20, 0.17)	0.845	0	0.402	0.976	0.152	0.421	0
ALP (U/L)	13	31	−0.06 (−0.26, 0.15)	0.598	0	0.195	0.894	0.613	0.309	0
Glucose (mg/dL)	20	54	0.17 (−0.14, 0.48)	0.285	71.03	<0.001	0.001	0.005	0.043	5
Creatinine (mg/dL)	14	38	0.04 (−0.20, 0.28)	0.727	34.69	<0.001	0.139	0.633	0.22	0
BUN (mg/dL)	29	73	−0.94 (−1.30, −0.58)	<0.001	82.32	<0.001	<0.001	<0.001	<0.001	2639

NS: number of studies; NC: number of comparisons between tannins and control treatment; WMD: weighted mean differences between treatments with tannins and control; CI: confidence interval of WMD; *p*-value to χ2 (Q) test of heterogeneity; I^2^: proportion of total variation of size effect estimates that is due to heterogeneity; Egger’s regression asymmetry test; Begg’s rank correlation method; NFs: Rosenberg’s fail-safe number; ALT: alanine aminotransferase; AST: aspartate aminotransferase; ALP: alkaline phosphatase; BUN: blood urea nitrogen.

### 3.3. Growth Performance, Carcass Characteristics, and Meat Composition

Dietary tannin in small ruminant diets decreased cold carcass weight (*p* = 0.032), but had no significant effect on FBW (*p* = 0.669), ADG (*p* = 0.698), FCR (*p* = 0.190), and HCW (*p* = 0.207) (Table 3). Dietary tannin decreased drip loss (*p* < 0.001) and subcutaneous fat thickness (WMD = −2.32) and increased WHC (*p* = 0.001). No significant effects were observed for L* (*p* = 0.515), a* (*p* = 0.383), b* (*p* = 0.947), WBSF (*p* = 0.360), or cooking loss (*p* = 0.394). No significant effects were observed for moisture (*p* = 0.397), protein (*p* = 0.682), ash (*p* = 0.331), or fat (*p* = 0.601) contents.

**Table 3:** The effects of dietary tannin on carcass characteristics and physicochemical parameters of meat of small ruminant.

	NS	NC	Tannins		Heterogeneity		Bias (*p*-Value)			NFs
			WMD (95% CI)	*p*- Value	I^2^ (%)	*p*- Value	Egger	Begg	NFs	
Carcass and growth characteristics										
Final body weight (kg)	46	112	0.04 (−0.14, 0.21)	0.669	65.82	<0.001	0.299	0.223	0.259	0
Average daily gain (g/day)	53	146	0.04 (−0.17, 0.25)	0.698	78.39	<0.001	<0.001	0.050	0.039	21
Feed conversion ratio	29	73	−0.14 (−0.35, 0.07)	0.190	59.17	<0.001	<0.001	0.841	0.007	93
Cold carcass weight (kg)	17	47	−0.27 (−0.53, −0.02)	0.032	62.95	<0.001	<0.001	<0.001	<0.001	155
Hot carcass weight (kg)	43	94	−0.14 (−0.34, 0.07)	0.207	73.13	<0.001	0.291	0.054	0.009	101
Subcutaneous fat thickness (mm)	8	24	−2.32 (−4.59, −0.06)	0.012	98.99	<0.001	<0.001	0.016	<0.001	249
Meat physicochemical characteristics										
24 h postmortem pH		79	0.22 (0.03, 0.41)	0.021	59.48	<0.001	<0.001	0.251	0.04	12
Water holding capacity (WHC, %)	8	20	0.39 (−0.51, 0.48)	0.001	6.19	0.336	0.693	0.725	<0.001	62
WBSF (kgf/cm^2^)	26	64	−0.43 (−1.35, 0.49)	0.36	97.45	<0.001	<0.001	0.05	<0.001	233
Cooking loss (%)	21	59	0.23 (−0.30, 0.77)	0.394	93.15	<0.001	0.001	0.326	0.006	80
Drip loss (%)	10	28	−0.46 (−0.69, −0.23)	<0.001	36.22	<0.001	<0.001	0.053	<0.001	256
Moisture (g/kg)	18	45	0.10 (−0.13, 0.31)	0.397	48.94	<0.001	<0.001	0.525	0.075	0
Proteins (g/kg)	19	47	0.06 (−0.21, 0.33)	0.682	66.42	<0.001	<0.001	0.582	0.196	0
Ash (g/kg)	18	47	0.44 (−0.44, 1.31)	0.331	96.38	<0.001	0.069	<0.001	<0.001	211
Fat (g/kg)	16	41	−0.12 (−0.55, 0.32)	0.601	84.13	<0.001	<0.001	0.189	0.168	0
Meat color										
Lightness (L*)	31	78	0.09 (−0.18, 0.35)	0.515	79.41	<0.001	<0.001	0.89	0.122	0
Redness (a*)	31	78	0.14 (−0.18, 0.46)	0.383	84.57	<0.001	0.017	0.085	0.014	63
Yellowness (b*)	31	78	−0.02 (−0.23, 0.37)	0.947	93.78	<0.001	0.107	0.938	0.063	0

NS: number of studies; NC: number of comparisons between tannins and control treatment; WMD: weighted mean differences between treatments with tannins and control; CI: confidence interval of WMD; *p*-value to χ2 (Q) test of heterogeneity; I^2^: proportion of total variation of size effect estimates that is due to heterogeneity; Egger’s regression asymmetry test; Begg’s rank correlation method; NFs: Rosenberg’s fail-safe number; WBSF: Warner-Bratzler shear force.

The fatty acid profile was improved by tannin inclusion in the diet of small ruminants (Table 4). Particularly, tannins increased C18:2 ω6 (*p* < 0.001), C18:3 ω3 (*p* < 0.001), C20:4 ω6 (*p* = 0.026), C20:5 ω3 (*p* = 0.021), and C22:5 ω3 (*p* < 0.001). Regarding the summaries, ratios, and indices, tannins increased ω3 FA (*p* < 0.001), ω6 FA (*p* < 0.001), total PUFA (*p* < 0.001), and ∆9C16 (*p* = 0.044), whereas no significant effects were observed for C18:0 (*p* = 0.139), C18:1 c9 (*p* = 0.065), C18:1 t11 (*p* = 0.168), C18:2 c9t11 (*p* = 0.308), C22:6 ω3 (*p* = 0.608), ω6: ω3 ratio (*p* = 0.356), total SFA (*p* = 0.287), total MUFA (*p* = 0.062), and ∆9C18 (*p* = 0.077).

**Table 4:** Effects of dietary tannin on the fatty acid profile of meat from small ruminants.

	NS	NC	Tannins		Heterogeneity		Bias (*p*-Value)			NFs
			WMD (95% CI)	*p*- Value	I^2^ (%)	*p*- Value	Egger	Begg	NFs	
Fatty acid profile (g/100 g FA)										
Stearic (C18:0)	19	44	−0.39 (−0.78, −0.01)	0.139	81.42	0.043	0.004	0.117	<0.001	252
Oleic (C18:1 c9)	19	43	−0.34 (−0.87, 0.19)	0.065	89.36	<0.001	<0.001	0.225	<0.001	281
trans-Vaccenic (C18:1 t11)	11	27	0.34 (−0.14, 0.83)	0.168	80.43	<0.001	0.001	0.021	0.003	50
Linoleic (LA, C18:2 ω6)	19	44	0.76 (0.45, 1.08)	<0.001	72.5	<0.001	0.246	0.044	<0.001	1268
Conjugated linoleic (CLA, C18:2 c9t11)	13	30	0.28 (−0.25, 0.80)	0.308	85.11	<0.001	0.097	0.357	0.009	34
α–Linolenic (ALA, C18:3 ω-3)	16	37	0.85 (0.47, 1.22)	<0.001	75.52	<0.001	<0.001	0.001	<0.001	918
Arachidonic (ARA, C20:4 ω6)	11	30	0.57 (0.07, 1.06)	0.026	83.83	<0.001	0.023	0.62	<0.001	289
Eicosapentaenoic (EPA, C20:5 n-3)	12	30	0.50 (0.08, 0.93)	0.021	79.57	<0.001	0.021	0.112	<0.001	224
Docosapentaenoic (DPA, C22:5 ω3)	12	33	0.62 (0.23, 1.01)	<0.001	75.89	<0.001	0.001	0.045	<0.001	420
Docosahexaenoic (DHA, C22:6 ω3)	9	23	0.12 (−0.34, 0.58)	0.608	78.17	<0.001	0.805	0.832	0.133	0
Total SFA (%)	17	40	−0.20 (−0.56, 0.17)	0.287	78.59	<0.001	0.748	0.284	0.009	45
Total MUFA (%)	17	40	−0.32 (−0.66, 0.02)	0.062	75.38	<0.001	0.121	0.204	<0.001	153
Total PUFA (%)	16	39	0.90 (0.59, 1.20)	<0.001	67.63	<0.001	<0.001	<0.001	<0.001	1363
Total ω3 (%)	11	27	0.72 (0.38, 1.05)	<0.001	63.87	<0.001	<0.001	0.007	<0.001	437
Total ω6 (%)	11	27	0.80 (0.46, 1.14)	<0.001	61.45	<0.001	<0.001	0.869	<0.001	377
ω6/ω3 ratio	10	23	0.22 (−0.24, 0.67)	0.356	78.49	<0.001	0.489	0.812	0.025	10
∆9C16	5	13	−0.56 (−1.11, −0.02)	0.044	69.04	<0.001	0.452	0.252	<0.001	51
∆9C18	5	13	−0.58 (−1.21, 0.06)	0.077	76.49	<0.001	0.77	0.858	<0.001	51

NS: number of studies; NC: number of comparisons between tannins and control treatment; WMD: weighted mean differences between treatments with tannins and control; CI: confidence interval of WMD; *p*-value to χ2 (Q) test of heterogeneity; I^2^: proportion of total variation of size effect estimates that is due to heterogeneity; Egger’s regression asymmetry test; Begg’s rank correlation method; NFs Rosenberg’s fail-safe number; SFA: saturated fatty acid; MUFA: monounsaturated fatty acid; PUFA: polyunsaturated fatty acid.

### 3.4. Meta-Regression and Publication Bias

As observed in Table 1, Table 2, Table 3 and Table 4, heterogeneity was high (>60%) for all parameters except for HCellD (*p* = 0.018, I^2^ = 40.66%), CellD (*p* = 0.039, I^2^ = 47.35%), butyrate (*p* = 0.002, I^2^ = 46.44%), hemoglobin (*p* = 0.732, I^2^ = 0%), total protein (*p* = 0.004, I^2^ = 34.12%), globulin (*p* = 0.755, I^2^ = 0%), albumin (*p* = 0.001, I^2^ = 42.75%), AST (*p* = 0.402, I^2^ = 0%), ALP (*p* = 0.195, I^2^ = 0%), and creatinine (*p* < 0.001, I^2^ = 34.69%) (Supplementary Table S2). Concerning carcass and meat quality, low heterogeneity was observed for fat (*p* < 0.001, I^2^ = 0%), drip loss (*p* < 0.001, I^2^ = 36.22%), and moisture (*p* < 0.001, I^2^ = 48.94%).

Following Egger’s test [28], publication bias existed for all intake and nitrogen metabolism parameters (*p* < 0.01), DMD (*p* < 0.001), NDFD (*p* = 0.001), CellD (*p* = 0.004), NH_3_-N (*p* = 0.031), acetate (*p* < 0.001), propionate (*p* < 0.001), acetate: propionate ratio (*p* < 0.001), hemoglobin (*p* = 0.011), glucose (*p* = 0.001), BUN (*p* < 0.001), ADG (*p* < 0.001), FCR (*p* < 0.001), CCW (*p* < 0.001), L* (*p* < 0.001), a* (*p* = 0.017), WBSF (*p* < 0.001), cooking loss (*p* = 0.001), drip loss (*p* < 0.001), moisture (*p* < 0.001), protein (*p* < 0.001), fat (*p* < 0.001), C18:0 (*p* = 0.004), C18:1 c9 (*p* < 0.001), C18:1 t11 (*p* = 0.001), C18:3 ω3 (*p* < 0.001), C20:4 ω6 (*p* = 0.023), C20:5 ω3 (*p* = 0.021), C22:5 ω3 (*p* = 0.001), total ω3 (*p* < 0.001), total ω6 (*p* < 0.001), and total PUFA (*p* < 0.001). However, according to Begg and Mazumdar [29], publication bias was not significant for any of the following variables: NDFI (*p* = 0.084), N intake (*p* = 0.057), retained N (*p* = 0.202), ADG (*p* = 0.050), FCR (*p* = 0.841), L* (*p* = 0.890), a* (*p* = 0.085), cooking loss (*p* = 0.326), drip loss (*p* = 0.053), moisture (*p* = 0.525), protein (*p* = 582), fat (*p* = 0.189), C18:0 (*p* = 0.117), C18:1 c9 (*p* = 0.225), C20:4 ω6 (*p* = 0.620), C20:5 ω3 (*p* = 0.112), or total ω6 (*p* = 0.869). For the rest of the variables where Rosenberg’s NFs was significant (*p* < 0.05), its values were 7314 (DMI), 376 (DMI-FI), 155 (CPI), 1285 (DMD), 2379 (NDFD), 437 (NH_3_-N), 796 (urinary N), 846 (N fecal), 2639 (BUN), 155 (CCW), WBSH (233), 918 (C18:3 ω3), 420 (C22:5 ω3), 437 (total ω3), and 1363 (total PUFA), and were higher than the Rosenthal NFs of 340 (5 × 66 + 10), 60 (5 × 10 + 10), 110 (5 × 20 + 10), 200 (5 × 38 + 10), 135 (5 × 25 + 10), 215 (5 × 41 + 10), 155 (5 × 29 + 10), 150 (5 × 28 + 10), 155 (5 × 29 + 10), 95 (5 × 17 + 10), 140 (5 × 26 + 10), 90 (5 × 16 + 10), 70 (5 × 12 + 10), 65 (5 × 11 + 10), and 90 (5 × 16 + 10), needed to declare the mean effect size significant—despite the possibility of no publication bias [7] for these parameters. Publication bias was reported only for hemoglobin, glucose, and C18:1 t11, where Rosenberg’s NFs of 50, 2, and 5, respectively, were lower than 155 (5 × 29 + 10), 280 (5 × 54 + 10), and 145 (5 × 27 + 10), respectively, required to declare publication bias.

### 3.5. Subgroup Analysis

**Figure 2:**
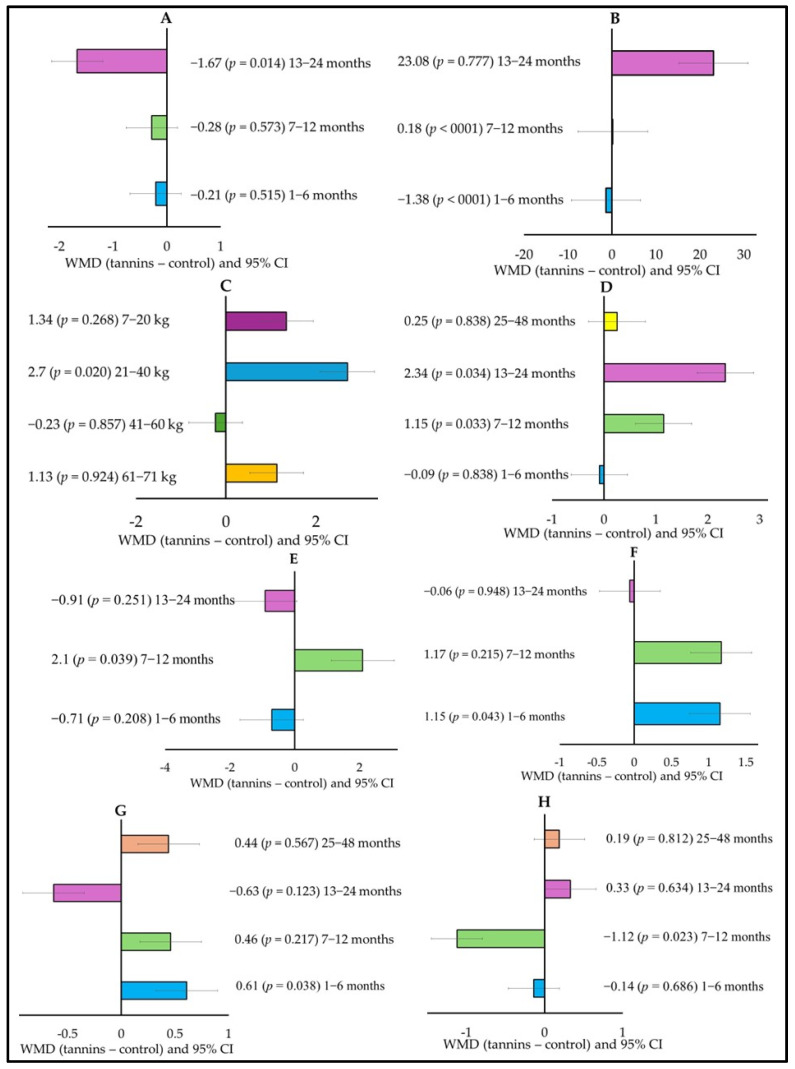
Subgroup analysis (subgroup = age of animals) of the effect of dietary tannins on the diet of small ruminants; WMD, weighted mean difference between tannin treatment and control. (**A**) Dry matter intake (DMI; g/day), (**B**) Crude protein intake (CPI; g/day), (**C**) Average daily gain (ADG; g/day), (**D**) Crude protein digestibility (CPD; g/kg DM), (**E**) Fecal N, (**F**) Retained N, (**G**) Ruminal pH, and (**H**) Acetate (mol/100 mol).

The effects of age on the responses of small ruminants to tannins are shown in Figure 2 (covariate = animal age). DMI decreased (WMD = −1.67; *p* = 0.014) when tannins were in the diet of animals aged 13–24 months (Figure 2A), whereas CPI increased (WMD = 0.18; *p* < 0001) when tannins were in the diets of animals aged 7–12 months, and decreased (WMD = −1.38, *p* < 0001) when tannins were in diets of animals aged 1–6 months (Figure 2B). A lower (WMD = −1.61; *p* = 0.035) ADG was observed in animals aged 13–24 months (Figure 2C), while CPD was higher for animals aged 7–12 months (WMD = 1.15; *p* = 0.033) and 13–24 months (WMD = 2.34; *p* = 0.034) (Figure 2D). The age of the animals also affected nitrogen metabolism, including fecal N, which was higher (WMD = 2.1; *p* = 0.039) for animals aged 7–12 months (Figure 2E), and retained N (WMD = 1.15; *p* = 0.043), which was higher for animals aged 1–6 months (Figure 2F). Fermentation parameters were also affected by age when tannins were present in small ruminants’ diet, where ruminal pH was higher (WMD = 0.61; *p* = 0.038) for animals aged 1–6 months (Figure 2G), and acetate was lower (WMD = −1.12; *p* = 0.023) in animals aged 7–12 months (Figure 2H).

**Figure 3:**
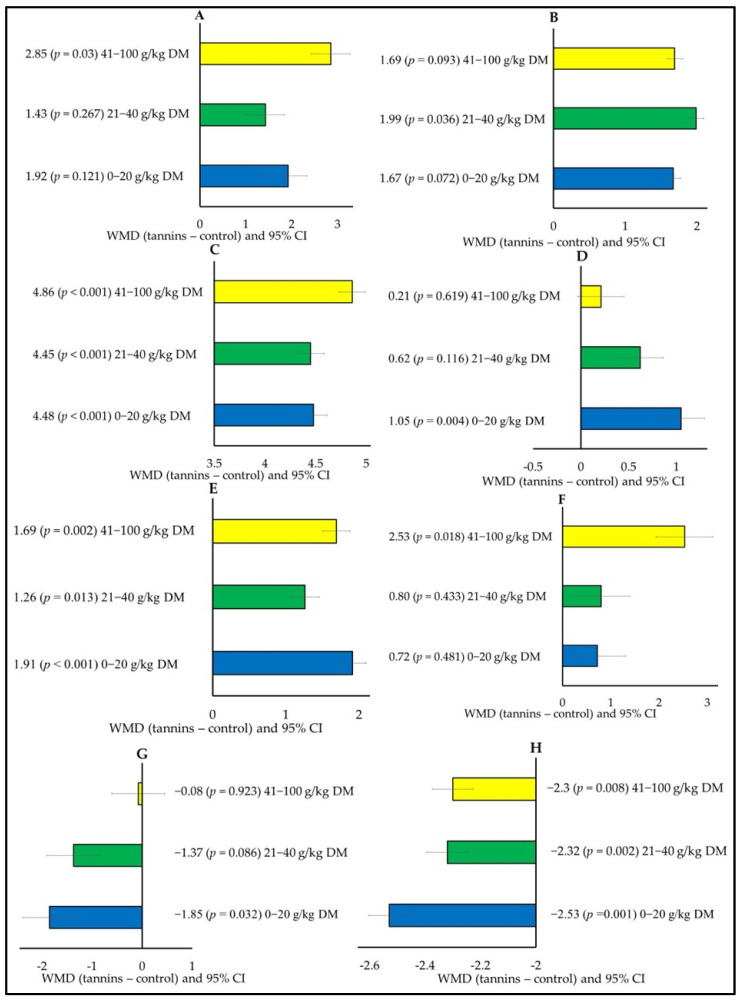
Subgroup analysis (subgroup = tannin concentration) of the effect of tannins on the diet of small ruminants; WMD, weighted mean difference between tannin treatment and control. (**A**) Crude protein intake (CPI; g/day), (**B**) Feed conversion ratio (FCR), (**C**) Dry matter digestibility (DMD; g/kg), (**D**) Final body weight (FBW, kg), (**E**) Hot carcass weight (HCW, kg), (**F**) α-linolenic acid (ALA, C18:3 ω3, g/100 g FA), (**G**) Arachidonic acid (ARA, C20:4 ω6, g/100 g FA), and (**H**) Docosahexaenoic acid (DHA, C22:6 ω3, g/100 g FA).

Figure 3 shows that CPI increased (WMD = 2.85; *p* = 0.03) only when tannins were supplemented at high concentration (41–100 g/kg DM; Figure 3A), whereas only moderate tannin concentration (21–40 g/kg) increased (WMD = 1.99; *p* = 0.036) FCR (Figure 3B). DMD increased with the three concentrations (WMD = 4.86; *p* < 0.001) for 41–100 g/kg DM, (WMD = 4.45; *p* < 0.001) for 21–40 g/kg DM, and (WMD = 4.48; *p* < 0.001) for 0–20 g/kg DM (Figure 3C). FBW increased (WMD = 1.05; *p* = 0.004) with low tannin supplementation (0–20 g/kg DM; Figure 3D), while HCW increased with the three tannin concentrations WMD = 1.69 (*p* = 0.002) for 41–100 g/kg DM, WMD = 1.26 (*p* = 0.013) for 21–40 g/kg DM, and WMD = 1.91 (*p* < 0.001) for 0–20 g/kg DM (Figure 3E). Tannin concentration in the diets also affected the FA profile. C18:3 ω3 increased (WMD = 2.53; *p* = 0.018) with high tannin supplementation (41–100 g/kg DM; Figure 3F), whereas C20:4 ω6 decreased (WMD = −1.85; *p* = 0.032) with low tannin supplementation (0–20 g/kg DM; Figure 3G). C22:2 ω6 decreased with the three tannin concentrations (WMD = −2.3; *p* = 0.008) for 41–100 g/kg DM, (WMD = −2.32; *p* = 0.002) for 21–40 g/kg DM, and (WMD = −2.53; *p* = 0.001) for 0–20 g/kg DM (Figure 3H).

**Figure 4:**
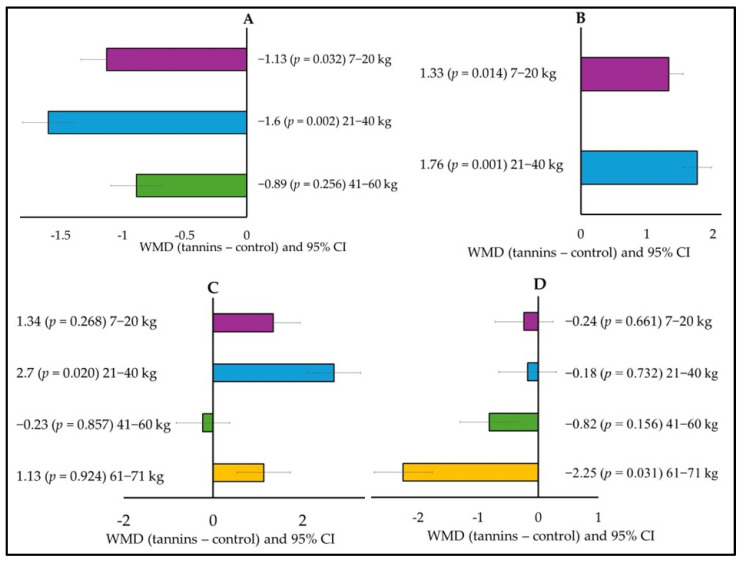
Subgroup analysis (subgroup = initial weight) of the effect of tannins on the diet of small ruminants; WMD, weighted mean difference between tannins treatment and control. (**A**) Average daily gain (ADG, g/day), (**B**) Feed conversion ratio (FCR), (**C**) N urine (g/day), and (**D**) Ruminal pH.

Subgroup analysis showed that the initial weight of the animals also affected their response to tannins (Figure 4). Tannin supplementation decreased ADG in animals weighed 7–20 kg (WMD = −1.13; *p* = 0.032) and 21–40 kg (WMD = −1.6; *p* = 0.002; Figure 4A), but the FCR increased for the same weight categories (WMD = 1.33; *p* = 0.014) for 7–20 kg and (WMD = 1.76; *p* = 0.001) for 21–40 kg (Figure 4B). Urinary N increased (WMD = 2.7; *p* = 0.020) in animals weighing 21–40 (Figure 4C), and ruminal pH decreased (WMD = −2.25; *p* = 0.031) in animals weighing 61–71 kg (Figure 4D).

**Figure 5:**
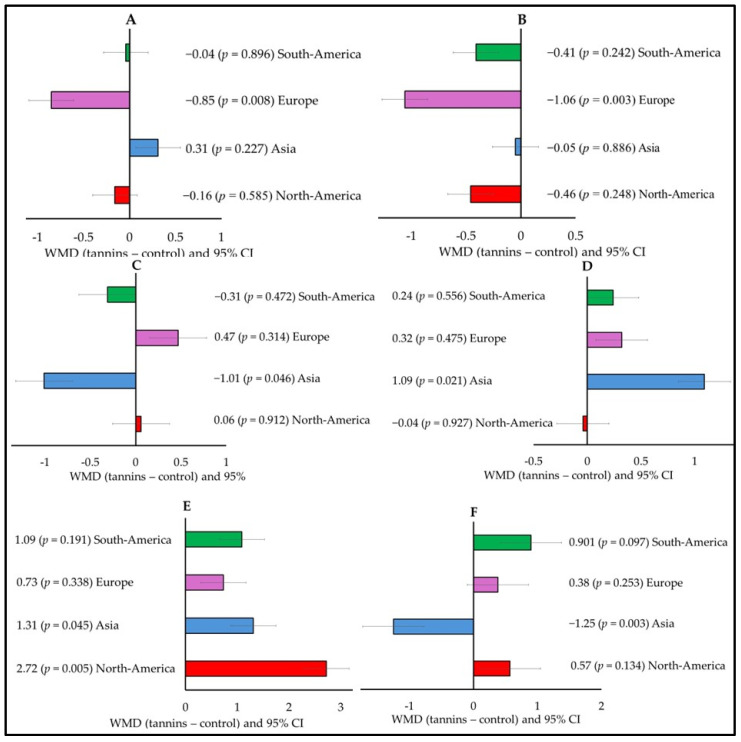
Subgroup analysis (subgroup = continent) of the effect of tannins on the diet of small ruminants; WMD, weighted mean difference between tannins treatment and control. (**A**) Final body weight (FBW, kg), (**B**) Hot carcass weight (HCW, kg), (**C**) Conjugated linoleic acid (CLA, C18:2 9tc11, g/100 g FA), (**D**) Meat pH, (**E**) N fecal (g/day), and (**F**) Acetate (mol/100 mol).

Growth performance and meat quality also varied according to the continent when small ruminants were supplied with tannins. In Europe, tannin supplementation decreased FBW (WMD = −0.85; *p* = 0.008; Figure 5A) and HCW (WMD = −1.06; *p* = 0.003; Figure 5B). When tannins were supplemented to small ruminants in Asia, there was a significant decrease in acetate (WMD = −1.25; *p* = 0.003; Figure 5F) and an increase in C18:1 c9t11 (WMD = 1.96; *p* = 0.006; Figure 5C), meat pH (WMD = 1.09; *p* = 0.021; Figure 5D), and fecal N (WMD = 1.31; *p* = 0.045; Figure 5E).

## 4. Discussion

### 4.1. Dry Matter Intake, Nutrient Digestibility, and Growth Performance

Tannins are a group of secondary metabolites present in plants as part of their defense mechanisms. These were initially considered anti-nutritional factors because of their effects on feed intake and digestibility [32]. In recent years, their integration into ruminant diets has emerged as a pivotal strategy for enhancing animal products and sustainability, given the increased challenges of climate change, land degradation, and higher demand for livestock products [33,34]. Some reviews have suggested that the astringent properties of tannins in ruminant diets may reduce feed intake [32]. However, this meta-analysis highlights that tannin supplementation improves DM and forage intake. The absence of negative impact might be due to the average tannins dose of 41.3 g/kg DM, which was lower than 50 g/kg DM, reported to negatively impact intake [35]. Contrary to our results on small ruminants, two previous meta-analyses conducted on dairy cows in production and beef cattle reported no significant impact on intake with tannins supplemented at 9.5 and 14.61 g kg^−1^ DM [35,36]. Overall, these results suggest that tannins can be incorporated into small ruminants and cattle without negatively affecting their feed intake.

Indeed, small ruminants are reported to not only withstand but also have a higher efficiency of valorizing tannin-containing feed than cattle. Small ruminants, especially goats, have naturally adapted as browsers and consume a wide range of vegetation, including shrubs and trees, which typically contain high tannin levels [37]. Several reviews have focused on tannin concentration as a probable factor of variation. While low levels have no impact on animals’ performances, moderate levels of tannins can improve protein utilization by protecting dietary proteins from excessive degradation in the rumen, and higher levels may lead to negative effects on nutrient absorption and animal performance owing to the formation of indigestible tannin-protein complexes [38]. However, in this meta-analysis, according to the subgroup analysis, DMI did not vary according to tannin concentration but was affected by the age of the animals. To avoid ingestion problems, tannin-containing diets should be avoided in animals older than 13 months. Goats have been reported to be more tolerant of tannins than sheep [37]. When evolved into tannin-rich pastures, goats had better feed and protein use efficiency and improved weight gain than sheep [37]. However, subgroup analysis revealed no significant difference between the DMI of sheep and goat species.

The absence of effects on nutrient intake (CP and NDF) could indicate the consumption of low-quality forage-containing tannins by small ruminants. Crude protein intake increased with higher concentrations of tannins in the diet (41–100 g/kg DM) and for younger animals (1–12 months). Younger animals have higher protein requirements for growth and development than older animals [39]. They may exhibit compensatory feeding behaviors, such as increasing their intake of tannin-rich feed to meet their protein needs. Based on the results obtained for nutrient intake and digestibility, a higher intake of DM may result in a shorter retention time of forage in the diet, leading to rapid rumen emptying, which potentially influences feed digestibility [40]. The decrease in digestibility of DM, CP, NDF, and ADF suggests that the primary effect of tannins in the rumen environment is due to their interaction with proteins, enzymes, and carbohydrates, rather than their astringent properties. Although tannins are known to alter certain protozoan species that are primary producers of butyrate, this meta-analysis verified that tannins increase the molar proportion of butyrate, which, according to Vogels et al. [41], can be associated with protozoa, facilitating the transfer of H_2_ to archaea through synergistic interactions between methanogens and ciliates. Contrary to our results, a meta-analysis conducted by Berça et al. [42] showed that CT increased the molar proportion of propionate, which was negatively correlated with CH_4_ production due to competition for H+. In the rumen, butyrate and acetate release H+, which is used by methanogens to produce CH_4_, whereas propionate competes with CH_4_ as an H+ sink [43].

### 4.2. Nitrogen Metabolism and Blood Metabolites

Ruminants have low efficiency in converting ingested proteins into animal products because a significant portion of this protein is wasted in the form of NH_3_-N in the rumen [44]. In a subsequent meta-analysis, dietary supplementation with tannins reduced protein degradability, which probably reduced rumen NH_3_-N concentration, which is usually absorbed into the bloodstream as BUN and excreted as urea in the urine [45]. Improved nitrogen utilization efficiency was expected from animals receiving dietary tannins owing to reduced protein degradability and probable outflow and absorption in the small intestine. However, the higher total nitrogen elimination through fecal N was probably due to the increased rumen-bypass proteins through their endogenous loss. The loss of nitrogen through fecal N cancels the positive impact expected from the bypass of ruminal proteins. However, shifting the excretion of nitrogen to feces instead of urine would benefit the environment. Nitrogen excreted via manure is less volatile than that from urine and has a lower chance of being converted to nitrous oxide, a greenhouse gas [46]. Therefore, in addition to its positive effect on productivity, tannin supplementation in small ruminants may be an interesting strategy to preserve the environment.

Subgroup analysis revealed that nitrogen use efficiency was mainly affected by the age of small ruminants. Young animals (1–6 months) showed high retained N despite the lower N intake, which testified to their higher N use efficiency compared to animals aged 7–12 months. In contrast, despite higher N intake for animals aged 7–12 months, these older animals had no significant effect on N retention but only increased fecal N and ruminal NH_3_-N release, which are major contributors to greenhouse gas emissions [47]. The higher N retention in young animals may be due to the increased need to utilize proteins to protect the intestinal epithelium against tannins. In addition to these defense mechanisms, animals may retain proteins, such as digestive enzymes, mucus, and salivary glycoproteins, to regenerate epithelial cells and compensate for endogenous losses in the gastrointestinal tract [48]. The higher rumen pH for the same age category (1–6 months) confirmed the following hypothesis:

### 4.3. Growth Performance and Meat Quality

In this meta-analysis, tannins did not affect ADG, FCR, HCW, or FBW, probably because the digestibility of proteins decreased in response to dietary tannins, despite compensation by higher intake. Thus, according to the following meta-analysis, despite the presence of tannins in the diet, small ruminants have low protein use efficiency. Similar results were reported in a meta-analysis of lactating cows having tannins [36]. In the subgroup analysis, a lower ADG was observed for 13 to 24-year-old animals fed tannins, which could be associated with the reduced DMI and CPI, despite the higher CP digestibility and lower NH_3_-N reported for these animals. Therefore, to better utilize tannin-containing feed, it would be better to avoid these diets for small ruminants older than 13–24 months due to their negative effects on productivity. The initial weight was also a determinant in the response to tannins, and lean animals (7–40 kg) showed lower ADG than animals weighing more than 41 kg. Indeed, smaller animals could have prioritized energy for maintenance over growth, especially because of low nutrient digestibility, which led to low availability of energy for weight gain. The concentration of tannins in the diet affected FBW, which increased significantly only at lower concentrations (0–20 g/kg DM). Thus, to avoid low small ruminant growth performance, the initial weight and age of animals should be considered when having tannin-based diets.

In contrast to the growth parameters, some physical quality characteristics were affected. In this meta-analysis, tannins were found to increase meat pH, which may explain the observed increase in water-holding capacity. According to Warner [49], meat with a higher pH has a better water retention capacity. Higher meat pH and WHC decrease the loss of soluble nutrients and prevent poor meat quality [50]. A decrease in drip loss was expected because of the strong negative correlation between drip loss and water-holding capacity [50]. Moreover, despite the higher water-holding capacity of the meat of small ruminants fed tannin-based diets, the moisture content was similar between the groups. Similarly, dietary tannin did not affect the nutritional parameters of meat, such as ash, protein, and fat.

Color and WBSF-related tenderness are the most important attributes that attract consumers and determine the quality of meat cuts [50]. Meat color is mainly affected by fat and the conversion of oxymyoglobin to metmyoglobin [51]. In particular, L* and a*, contrary to other meta-analyses [52], were not significantly increased by dietary tannin. Several studies have reported a positive correlation between b* and fat content in meat, and the absence of an effect of tannins on b* can be explained by their absence in meat fat. Increased tenderness can be attributed to the production of reactive oxygen species (ROS), which increase the degradation of toughness-related structural proteins [53]. The improvement in antioxidant activity by helping animals fight ROS usually increases the rate of collagen turnover [54] or postmortem proteolysis of cytoskeletal proteins, which is mediated by calpain enzymes when animals are exposed to ROS [55]. In this meta-analysis, the absence of improvement in antioxidant activity following dietary tannin could explain the lower WBSF, indicating functional and structural damage caused by ROS in muscle cells and tissues.

### 4.4. Meat Fatty Acid Profile

In addition to their effects on the physical and chemical properties of meat, tannins have a profound influence on the fatty acid profile. In the rumen, *Butyrivibrio fibrisolvens* converts linoleic acid (LA, C18:2 ω6) to rumenic acid (an isomer of C18:2 c9t11) and rumenic acid to vaccenic acid (C18:1 t11), whereas *Butyrivibrio proteoclasticus* converts vaccenic acid to stearic acid [53]. Interestingly, in this meta-analysis, the inclusion of tannins in the diet of small ruminants probably acted directly on *Butyrivibrio fibrisolvens* and inhibited the first step of biohydrogenation, leading to enhanced linoleic acid. Some studies suggest that high dietary intake of LA has been linked to reduced cardiovascular risk factors and improved long-term insulin resistance [56]. LA intake may have adverse pro-inflammatory effects in humans [57].

Moreover, our meta-analysis reported increased concentrations of α-linolenic, arachidonic, eicosapentaenoic, docosapentaenoic, docosahexaenoic, total ω3, and total PUFA in the meat of small ruminants fed tannins. Epidemiological studies have associated the dietary benefits of ω3 FA, including α-linolenic acid, EPA, and DHA, with reduced cardiovascular disease and cancer risk [58]. However, the degree to which tannins affect this process varies depending on their concentration and the continent on which they are added. Subgroup analysis highlighted that meat containing increased C18:3 ω3 content is obtained with high levels of dietary tannins (41–100 g/kg DM), while total ω3 content was higher in Europe. These results could be due to recent efforts to fortify food in Europe with ω3 fatty acids [59], in addition to the adopted regulation number 116/2010, classifying and organizing food as a source of high ω3 fatty acid content [60].

In contrast, in the endoplasmic reticulum membrane, fatty acids bypassed from the diet and synthesized de novo are modified by desaturation and elongation. Palmitoleic acid (C16:1 c9) and oleic acid (C18:1 c9) are the major fatty acids in cells and are synthesized from palmitic and stearic acids, respectively, by the Δ9 desaturase enzyme (SCD gene) [61]. This enzyme is primarily involved in the synthesis of MUFA and PUFA from SFA, primarily C18:1 c9 from C18:0 and C18:1 c9t11 from C18:1 t11 [61]. In subgroup analysis, C18:1 c9t11 levels were higher when tannins were administered to small ruminants in Asia. This could be due to efforts to enhance the proportion of the most desired C18:1 c9t11 isomer, rumenic acid, by supplementing the C18:1 c9t11 precursor with vaccenic acid [62]. CLA has been associated with antiatherogenic, antidiabetogenic, anticarcinogenic, and immunomodulatory properties [63].

In this meta-analysis, the absence of the tannin effect on ∆9 desaturase explains the absence of variation in C18:0, C18:1 c9, C18:1 t11, C18:2 c9t11, total SFA, and total MUFA. Due to the absence of a desaturation metabolic pathway, the increase in PUFA is mainly due to the inhibition of ruminal biohydrogenation.

## 5. Conclusions

The results of this meta-analysis indicate that dietary tannins improved dry matter intake, decreased nutrient digestibility, and had no impact on growth performance. However, tannins affected nitrogen metabolism and increased fecal N at the expense of urinary N, especially in North America, and the retained N was higher for younger animals (1–6 months). Blood parameters did not vary, except for the observed decrease in blood urea nitrogen. Despite what has been reported in previous reviews, subgroup analysis showed no significant difference between goats and sheep in their response to tannins. The best results for feed conversion ratio were achieved for animals with small initial weights (7–40 kg). The final body weight increased with low tannin concentration (0–20 g/kg DM). Concerning meat quality, tannins did not affect chemical (meat pH, moisture, protein, ash, and fat) or physical (color (L*, a*, and b*), tenderness, cooking loss, WHC, except drip loss) parameters. However, tannin supplementation enhanced the fatty acid profile by increasing C18:2 ω6 and ω3 PUFA (ALA, EPA, DPA, and DHA), ARA, and PUFA. ALA was improved by a high dietary tannin concentration (41–100 g/kg DM), while a lower tannin concentration (0–20 g/kg DM) decreased ARA. Moreover, the FA profile varied according to continent, and higher ω3 PUFA were found in Europe, whereas C18:1 c9t11 was higher in Asia. To optimize the utilization of tannins, these factors should be considered when formulating diets for small ruminants.

## Data Availability

The raw data supporting the conclusions of this article will be made available by the authors without undue reservation.

## Supplementary Materials

The following supporting information can be downloaded at: https://www.mdpi.com/article/10.3390/ani15040596/s1, Table S1. Summary of the studies included in the meta-analysis; Table S2. Meta-regression of the covariate effect on weighted mean differences (WMD) between tannin and control treatments on diet nutrient intake and digestibility, fermentation, blood parameters, carcass characteristics, meat quality, and fatty acid profile. The adjusted R^2^ is the proportion of between-study variance (heterogeneity) explained by the covariate. References [37,64,65,66,67,68,69,70,71,72,73,74,75,76,77,78,79,80,81,82,83,84,85,86,87,88,89,90,91,92,93,94,95,96,97,98,99,100,101,102,103,104,105,106,107,108,109,110,111,112,113,114,115,116,117,118,119,120,121,122,123,124,125,126,127,128,129,130,131,132,133,134,135,136,137,138,139,140,141,142,143,144,145,146,147,148,149,150,151,152,153,154,155,156,157,158,159] are cited in the supplementary materials.

## References

[1] Joy A., Dunshea F.R., Leury B.J., Clarke I.J., DiGiacomo K., Chauhan S.S. (2020). Resilience of small ruminants to climate change and increased environmental temperature: A review. Animals.

[2] El Aich A., Rouzi M., Fernandez-Gimenez M., Alados C.L. (2022). The Degradation of Rangelands in the Middle Atlas During the Last Decades.

[3] Boukrouh S., Bouazzaoui Y., El Aich A., Mahyou H., Chikhaoui M., Ait Lafkih M., N’Dorma O., Alados C.L. (2024). Estimation of standing crop biomass in rangelands of the Middle Atlas mountains using remote sensing data. Afr. J. Range Forage Sci..

[4] Boukrouh S., Noutfia A., Moula N., Avril C., Louvieaux J., Hornick J.L., Chentouf M., Cabaraux J.F. (2023). Ecological, morpho-agronomical, and nutritional characteristics of *Sulla flexuosa* (L.) Medik. ecotypes. Sci. Rep..

[5] Boukrouh S., Noutfia A., Moula N., Avril C., Louvieaux J., Hornick J.L., Cabaraux J.F., Chentouf M. (2023). Ecological, morpho-agronomical, and bromatological assessment of sorghum ecotypes in Northern Morocco. Sci. Rep..

[6] Boukrouh S., Noutfia A., Moula N., Avril C., Louvieaux J., Hornick J.-L., Chentouf M., Cabaraux J.-F. (2024). Characterisation of bitter vetch (*Vicia ervilia* (L.) Willd) ecotypes: An ancient and promising legume. Exp. Agric..

[7] Malenica D., Kass M., Bhat R. (2022). Sustainable management and valorization of agri-food industrial wastes and by-products as animal feed: For ruminants, non-ruminants and as poultry feed. Sustainability.

[8] Shnawa H.A., Khalaf M.N., Taobi A.A.H., Panampilly B., Thomas S. (2020). General and Chemical Perspectives and Studies on Tannins as Natural Phenolic Compounds for Some Ecoefficient Applications. Natural Products Chemistry.

[9] Min B.R., Solaiman S., Waldrip H.M., Parker D., Todd R.W., Brauer D. (2020). Dietary mitigation of enteric methane emissions from ruminants: A review of plant tannin mitigation options. Anim. Nutr..

[10] Gardiner C.A., Clough T.J., Cameron K.C., Di H.J., Edwards G.R., de Klein C.A.M. (2016). Potential for forage diet manipulation in New Zealand pasture ecosystems to mitigate ruminant urine derived N_2_O emissions: A review. N. Z. J. Agric. Res..

[11] Retallick K.M., Faulkner D.B., Rodriguez-Zas S.L., Nkrumah J.D., Shike D.W. (2013). Relationship among performance, carcass, and feed efficiency characteristics, and their ability to predict economic value in the feedlot. J. Anim. Sci..

[12] Costa E.I.D.S., Ribiero C., Silva T.M., Ribeiro R.D.X., Vieira J.F., Lima A.G.V.D.O., Barbosa A.M., da Silva Júnior J.M., Bezerra L.R., Oliveira R.L. (2021). Intake, nutrient digestibility, nitrogen balance, serum metabolites and growth performance of lambs supplemented with *Acacia mearnsii* condensed tannin extract. Anim. Feed Sci. Technol..

[13] Brunetto A.L.R., dos Santos A.L.F., Zago I., Deolino G.L., Nora L., Molosse V.L., Lago R.V.P., Machado A.D.C., Wagner R., Nauderer J.N. (2024). Intake of Condensed Tannins (*Acacia mearnsii*) by Lambs in Confinement and Its Impact on Growth Performance, Rumen Environment, and Meat. Fermentation.

[14] Terra-Braga M., Poli C.H.E.C., Tontini J.F., Ahsin M., Van Vliet S., Villalba J.J. (2024). Trade-Offs between Selection of Crude Protein and Tannins in Growing Lambs. J. Anim. Sci..

[15] Ribeiro S.D.S., Vedovatto M., Palmer E.A., Franco G.L. (2024). Effects of *Acacia mearnsii* de Wild. Extract and Monensin on Intake, Digestibility, and Ruminal Variables of Lambs. Rev. Bras. Zootec..

[16] Gao C., Qi M., Zhou Y. (2024). Chestnut Tannin Extract Modulates Growth Performance and Fatty Acid Composition in Finishing Tan Lambs by Regulating Blood Antioxidant Capacity, Rumen Fermentation, and Biohydrogenation. BMC Vet. Res..

[17] Hughes J.M., Oiseth S.K., Purslow P.P., Warner R.D. (2014). A structural approach to understanding the interactions between colour, water-holding capacity and tenderness. Meat Sci..

[18] Purba R.A.P., Paengkoum P., Paengkoum S. (2020). The links between supplementary tannin levels and conjugated linoleic acid (CLA) formation in ruminants: A systematic review and meta-analysis. PLoS ONE.

[19] Kelln B.M., Penner G.B., Acharya S.N., McAllister T.A., Lardner H.A. (2020). Impact of condensed tannin-containing legumes on ruminal fermentation, nutrition, and performance in ruminants: A review. Can. J. Anim. Sci..

[20] Silanikove N., Tagari H., Shkolnik A. (1993). Comparison of rate of passage, fermentation rate and efficiency of digestion of high fiber diet in desert Bedouin goats compared to Swiss Saanen goats. Small Rumin. Res..

[21] Besharati M., Maggiolino A., Palangi V., Kaya A., Jabbar M., Eseceli H., De Palo P., Lorenzo J.M. (2022). Tannin in Ruminant Nutrition: Review. Molecules.

[22] Brooker J.D., O’donovan L.A., Skene I., Clarke K., Blackall L., Muslera P. (1994). *Streptococcus caprinus* sp. nov., a tannin-resistant ruminal bacterium from feral goats. Lett. Appl. Microbiol..

[23] Paul J., Barari M. (2022). Meta-analysis and traditional systematic literature reviews—What, why, when, where, and how?. Psychol. Mark..

[24] O’Dea R.E., Lagisz M., Jennions M.D., Koricheva J., Noble D.W.A., Parker T.H., Gurevitch J., Page M.J., Stewart G., Moher D. (2021). Preferred reporting items for systematic reviews and meta-analyses in ecology and evolutionary biology: A PRISMA extension. Biol. Rev..

[25] DerSimonian R., Laird N. (1986). Meta-analysis in clinical trials. Control. Clin. Trials.

[26] Deeks J.J., Higgins J.P.T., Altman D.G., Group C.S.M. (2019). Analysing data and undertaking meta-analyses. Cochrane Handb. Syst. Rev. Interv..

[27] Grant J., Hunter A. (2006). Measuring inconsistency in knowledgebases. J. Intell. Inf. Syst..

[28] Egger M., Smith G.D., Schneider M., Minder C. (1997). Bias in meta-analysis detected by a simple, graphical test. BMJ.

[29] Begg C.B., Mazumdar M. (1994). Operating characteristics of a rank correlation test for publication bias. Biometrics.

[30] Rosenberg M.S., Adams D.C., Gurevitch J. (1997). MetaWin: Statistical Software for Meta-Analysis with Resampling Tests.

[31] Littell J.H., Corcoran J., Pillai V. (2008). Systematic Reviews and Meta-Analysis.

[32] Salim R., Nehvi I.B., Mir R.A., Tyagi A., Ali S., Bhat O.M. (2023). A review on anti-nutritional factors: Unraveling the natural gateways to human health. Front. Nutr..

[33] Boukrouh S., Noutfia A., Moula N., Avril C., Hornick J.-L., Chentouf M., Cabaraux J.-F. (2023). Effects of *Sulla flexuosa* Hay as Alternative Feed Resource on Goat’s Milk Production and Quality. Animals.

[34] Boukrouh S., Noutfia A., Moula N., Avril C., Louvieaux J., Hornick J.-L., Cabaraux J.-F., Chentouf M. (2024). Growth Performance, Carcass Characteristics, Fatty Acid Profile, and Meat of Male Goat Kids Supplemented by Alternative Feed Resources: Bitter and Sorghum Grains. Arch. Anim. Breed..

[35] Orzuna-Orzuna J.F., Dorantes-Iturbide G., Lara-Bueno A., Mendoza-Martínez G.D., Miranda-Romero L.A., Hernández-García P.A. (2021). Effects of Dietary Tannins’ Supplementation on Growth Performance, Rumen Fermentation, and Enteric Methane Emissions in Beef Cattle: A Meta-Analysis. Sustainability.

[36] Herremans S., Vanwindekens F., Decruyenaere V., Beckers Y., Froidmont E. (2020). Effect of dietary tannins on milk yield and composition, nitrogen partitioning and nitrogen use efficiency of lactating dairy cows: A meta-analysis. J. Anim. Physiol. Anim. Nutr..

[37] Yisehak K., Kibreab Y., Taye T., Ribeiro Alves Lourenço M., Janssens G.P.J. (2016). Response to dietary tannin challenges in view of the browser/grazer dichotomy in an Ethiopian setting: Bonga sheep versus Kaffa goats. Trop. Anim. Health Prod..

[38] Min B.R., Solaiman S. (2018). Comparative aspects of plant tannins on digestive physiology, nutrition and microbial community changes in sheep and goats: A review. J. Anim. Physiol. Anim. Nutr..

[39] Wang D., Zhou L., Zhou H., Hou G., Li M., Shi L., Huang X., Guan S. (2014). Effects of nutrition level of concentrate-based diets on growth performance and carcass characteristics of Hainan black goats. Trop. Anim. Health Prod..

[40] Frutos P., Hervás G., Giráldez F.J., Mantecón A.R. (2004). Tannins and ruminant nutrition, Review. Span. J. Agric. Res..

[41] Vogels G.D., Hoppe W.F., Stumm C.K. (1980). Association of methanogenic bacteria with rumen ciliates. Appl. Environ. Microbiol..

[42] Berça A.S., Tedeschi L.O., da Silva Cardoso A., Reis R.A. (2023). Meta-analysis of the relationship between dietary condensed tannins and methane emissions by cattle. Anim. Feed Sci. Technol..

[43] Patra A., Park T., Kim M., Yu Z. (2017). Rumen methanogens and mitigation of methane emission by anti-methanogenic compounds and substances. J. Anim. Sci. Biotechnol..

[44] Zurak D., Kljak K., Aladrović J. (2023). Metabolism and utilisation of non-protein nitrogen compounds in ruminants: A review. J. Cent. Eur. Agric..

[45] Tan P., Liu H., Zhao J., Gu X., Wei X., Zhang X., Ma N., Johnston L.J., Bai Y., Zhang W. (2021). Amino acids metabolism by rumen microorganisms: Nutrition and ecology strategies to reduce nitrogen emissions from the inside to the outside. Sci. Total Environ..

[46] Patra A.K., Saxena J. (2011). Exploitation of dietary tannins to improve rumen metabolism and ruminant nutrition. J. Sci. Food Agric..

[47] Wang Y., Li X., Yang J., Tian Z., Sun Q., Xue W., Dong H. (2018). Mitigating greenhouse gas and ammonia emissions from beef cattle feedlot production: A system meta-analysis. Environ. Sci. Technol..

[48] Addisu S. (2016). Effect of dietary tannin source feeds on ruminal fermentation and production of cattle; a review. J. Anim. Feed Res..

[49] Warner R.D. (2023). The eating quality of meat: IV—Water holding capacity and juiciness. Lawrie’s Meat Science.

[50] Geletu U.S., Usmael M.A., Mummed Y.Y., Ibrahim A.M. (2021). Quality of Cattle Meat and Its Compositional Constituents. Vet. Med. Int..

[51] Tushar Z.H., Rahman M.M., Hashem M.A. (2023). Metmyoglobin reducing activity and meat color: A review. Meat Res..

[52] Orzuna-orzuna J.F., Dorantes-iturbide G., Lara-bueno A., Miranda-romero L.A. (2021). Growth Performance, Meat Quality and Antioxidant Status of Sheep Supplemented with Tannins: A Meta-Analysis. Animals.

[53] Malheiros J.M., Braga C.P., Grove R.A., Ribeiro F.A., Calkins C.R., Adamec J., Chardulo L.A.L. (2019). Influence of oxidative damage to proteins on meat tenderness using a proteomics approach. Meat Sci..

[54] Archile-Contreras A.C., Purslow P.P. (2011). Oxidative stress may affect meat quality by interfering with collagen turnover by muscle fibroblasts. Int. Food Res..

[55] Montgomery T., Leheska J. (2008). Effects of Various Management Practices on Beef-Eating Quality.

[56] Marangoni F., Agostoni C., Borghi C., Catapano A.L., Cena H., Ghiselli A., La Vecchia C., Lercker G., Manzato E., Pirillo A. (2019). Dietary linoleic acid and human health: Focus on cardiovascular and cardiometabolic effects. Atherosclerosis.

[57] Monnard C.R., Dulloo A.G. (2021). Polyunsaturated fatty acids as modulators of fat mass and lean mass in human body composition regulation and cardiometabolic health. Obes. Rev..

[58] Shahidi F., Ambigaipalan P. (2018). Omega-3 polyunsaturated fatty acids and their health benefits. Annu. Rev. Food Sci. Technol..

[59] Panse Manohar L., Phalke S.D., Hegde Mahabaleshwar V., Zanwar A.A., Adekar S.P. (2016). Fortification of Food with Omega-3 Fatty Acids. Omega-3 Fatty Acids: Keys to Nutritional Health.

[60] Regulation U.E. (2010). No 116/2010 of 9 February 2010 amending Regulation (EC) No 1924/2006 of the European Parliament and of the Council with regard to the list of nutrition claims. Off. J. Eur. Union L.

[61] Urrutia O., Mendizabal J.A., Alfonso L., Soret B., Insausti K., Arana A. (2020). Adipose Tissue Modification through Feeding Strategies and Their Implication on Adipogenesis and Adipose Tissue Metabolism in Ruminants. Int. J. Mol. Sci..

[62] Jaturasitha S., Chaiwang N., Kayan A., Kreuzer M. (2016). Nutritional strategies to improve the lipid composition of meat, with emphasis on Thailand and Asia. Meat Sci..

[63] Park Y., Park Y. (2009). Conjugated nonadecadienoic acid is more potent than conjugated linoleic acid on body fat reduction. J. Nutr. Biochem..

[64] Abdalla Filho A.L., Dineshkumar D., Barreal M., McManus C., Vasconcelos V.R., Abdalla A.L., Louvandini H. (2017). Performance, metabolic variables and enteric methane production of Santa Inês hair lambs fed *Orbignya phalerata* and *Combretum leprosum*. J. Anim. Physiol. Anim. Nutr..

[65] Abdalla Filho A.L., Corrêa P.S., Lemos L.N., Dineshkumar D., Issakowicz J., Ieda E.H., Lima P.M.T., Barreal M., McManus C., Mui T.S. (2017). Diets based on plants from Brazilian Caatinga altering ruminal parameters, microbial community and meat fatty acids of Santa Inês lambs. Small Rumin. Res..

[66] Abarghuei M.J., Rouzbehan Y., Alipour D. (2010). The influence of the grape pomace on the ruminal parameters of sheep. Livest. Sci..

[67] Abdullah M.A.M., Farghaly M.M., Youssef I.M.I. (2018). Effect of feeding Acacia nilotica pods to sheep on nutrient digestibility, nitrogen balance, ruminal protozoa and rumen enzymes activity. J. Anim. Physiol. Anim. Nutr..

[68] Adejoro F.A., Hassen A., Akanmu A.M., Morgavi D.P. (2020). Replacing urea with nitrate as a non-protein nitrogen source increases lambs’ growth and reduces methane production, whereas acacia tannin has no effect. Anim. Feed Sci. Technol..

[69] Aghamohamadi N., Hozhabri F., Alipour D. (2014). Effect of oak acorn (*Quercus persica*) on ruminal fermentation of sheep. Small Rumin. Res..

[70] Al Dobaib S.N. (2009). Effect of different levels of quebracho tannin on nitrogen utilization and growth performance of Najdi sheep fed alfalfa (*Medicago sativa*) hay as a sole diet. Anim. Sci. J..

[71] Animut G., Puchala R., Goetsch A.L., Patra A.K., Sahlu T., Varel V.H., Wells J. (2008). Methane emission by goats consuming different sources of condensed tannins. Anim. Feed Sci. Technol..

[72] Archimède H., Rira M., Barde D.J., Labirin F., Marie-Magdeleine C., Calif B., Périacarpin F., Fleury J., Rochette2 Y., Morgavi D.P. (2016). Potential of tannin-rich plants, *Leucaena leucocephala*, *Glyricidia sepium* and *Manihot esculenta*, to reduce enteric methane emissions in sheep. J. Anim. Physiol. Anim. Nutr..

[73] Balehegn M., Eik L.O., Tesfay Y. (2014). Replacing commercial concentrate by *Ficus thonningii* improved productivity of goats in Ethiopia. Trop. Anim. Health Prod..

[74] Ban C., Paengkoum S., Yang S., Tian X., Thongpea S., Purba R.A.P., Paengkoum P. (2022). Feeding meat goats mangosteen (*Garcinia mangostana* L.) peel rich in condensed tannins, flavonoids, and cinnamic acid improves growth performance and plasma antioxidant activity under tropical conditions. J. Appl. Anim. Res..

[75] Bandeira P.A.V., Filho J.M.P., de Azevêdo Silva A.M., Cezar M.F., Bakke O.A., Silva U.L., Borburema J.B., Bezerra L.R. (2017). Performance and carcass characteristics of lambs fed diets with increasing levels of *Mimosa tenuiflora* (Willd.) hay replacing Buffel grass hay. Trop. Anim. Health Prod..

[76] Batchu P., Hazard T., Lee J.H., Terrill T.H., Kouakou B., Kannan G. (2021). High-Condensed Tannin Diet and Transportation Stress in Goats: Effects on Physiological Responses, Gut Microbial Counts and Meat Quality. Animals.

[77] Salem HBen Makkar H.P.S., Nefzaoui A., Hassayoun L., Abidi S. (2005). Benefit from the association of small amounts of tannin-rich shrub foliage (*Acacia cyanophylla* Lindl.) with soya bean meal given as supplements to Barbarine sheep fed on oaten hay. Anim. Feed Sci. Technol..

[78] Chanjula P., Wungsintaweekul J., Chiarawipa R., Rugkong A., Khonkhaeng B., Suntara C., Cherdthong A. (2022). Effect of Feed Supplement Containing Dried Kratom Leaves on Apparent Digestibility, Rumen Fermentation, Serum Antioxidants, Hematology, and Nitrogen Balance in Goats. Fermentation.

[79] Chikwanha O.C., Muchenje V., Nolte J.E., Dugan M.E.R., Mapiye C. (2019). Grape pomace (*Vitis vinifera* L. cv. Pinotage) supplementation in lamb diets: Effects on growth performance, carcass and meat quality. Meat Sci..

[80] Chikwanha O.C., Raffrenato E., Muchenje V., Nolte J.V.E., Mapiye C. (2019). Effect of grape (*Vitis vinifera* L. cv. Pinotage) pomace supplementation on nutrient utilization in finisher lambs. Small Rumin. Res..

[81] Costa E.I.D.S., Ribeiro C.V.D.M., Silva T.M., Batista A.S.M., Vieira J.F., Barbosa A.M., da Silva Júnior J.M., Bezerra L.R., Pereira E.S., Oliveira R.L. (2021). Effect of dietary condensed tannins inclusion from *Acacia mearnsii* extract on the growth performance, carcass traits and meat quality of lambs. Livest. Sci..

[82] Dawson J.M., Buttery P.J., Jenkins D., Wood C.D., Gill M. (1999). Effects of dietary quebracho tannin on nutrient utilisation and tissue metabolism in sheep and rats. J. Sci. Food Agric..

[83] Dentinho M.T.P., Belo A.T., Bessa R.J.B. (2014). Digestion, ruminal fermentation and microbial nitrogen supply in sheep fed soybean meal treated with *Cistus ladanifer* L. tannins. Small Rumin. Res..

[84] Dey A., Dutta N., Sharma K., Pattanaik A.K. (2008). Effect of dietary inclusion of *Ficus infectoria* leaves as a protectant of proteins on the performance of lambs. Small Rumin. Res..

[85] El-Meccawi S., Kam M., Brosh A., Degen A.A. (2008). Heat production and energy balance of sheep and goats fed sole diets of *Acacia saligna* and *Medicago sativa*. Small Rumin. Res..

[86] Emami A., Fathi Nasri M.H., Ganjkhanlou M., Rashidi L., Zali A. (2015). Dietary pomegranate seed pulp increases conjugated-linoleic and -linolenic acids in muscle and adipose tissues of kid. Anim. Feed Sci. Technol..

[87] Emami A., Nasri M.H.F., Ganjkhanlou M., Zali A., Rashidi L. (2015). Effects of dietary pomegranate seed pulp on oxidative stability of kid meat. Meat Sci..

[88] Emami A., Ganjkhanlou M., Fathi Nasri M.H., Zali A., Rashidi L. (2015). Pomegranate seed pulp as a novel replacement of dietary cereal grains for kids. Small Rumin. Res..

[89] Fernandes J., Pereira Filho J., Menezes D., Caldas A.C., Cavalcante I., Oliveira J., Oliveira R., Júnior J.S., Cézar M., Bezerra L. (2021). Carcass and meat quality in lambs receiving natural tannins from *Mimosa tenuiflora* hay. Small Rumin. Res..

[90] Froutan E., Azizi O., Sadeghi G., Fatehi F., Lashkari S. (2014). Effects of different concentrations of ground oak acorn on growth performance, blood parameters and carcass characteristics of goat kids. Anim. Prod. Sci..

[91] Galvão J.M., Silva T.M., Silva W.P., Pimentel P.R.S., Barbosa A.M., Nascimento T.V.C., Lima A.G.V.O., Bezerra L.R., Oliveira R.L. (2020). Intake, digestibility, ingestive behavior, and nitrogen balance of goats fed with diets containing residue from tamarind fruit. Trop. Anim. Health Prod..

[92] García-Salas A., Bárcena-Gama J.R., Hernández-Sánchez D., Cobos-Peralta M.A., González-Muñoz S.S., Vaquera-Huerta H., Arias-Margarito L. (2022). Fattening performance and carcass characteristics of lambs supplemented with condensed tannins from *Acacia mearnsii* extract. S. Afr. J. Anim. Sci..

[93] Guerreiro O., Alves S.P., Soldado D., Cachucho L., Almeida J.M., Francisco A., Santos-Silva J., Bessa R.J.B., Jerónimo E. (2020). Inclusion of the aerial part and condensed tannin extract from *Cistus ladanifer* L. in lamb diets—Effects on growth performance, carcass and meat quality and fatty acid composition of intramuscular and subcutaneous fat. Meat Sci..

[94] Ghaffari M.H., Tahmasbi A.-M., Khorvash M., Naserian A.-A., Ghaffari A.H., Valizadeh H. (2014). Effects of pistachio by-products in replacement of alfalfa hay on populations of rumen bacteria involved in biohydrogenation and fermentative parameters in the rumen of sheep. J. Anim. Physiol. Anim. Nutr..

[95] Giller K., Sinz S., Messadene-Chelali J., Marquardt S. (2021). Maternal and direct dietary polyphenol supplementation affect growth, carcass and meat quality of sheep and goats. Animal.

[96] Girard M., Dohme-Meier F., Silacci P., Ampuero Kragten S., Kreuzer M., Bee G. (2016). Forage legumes rich in condensed tannins may increase n-3 fatty acid levels and sensory quality of lamb meat. J. Sci. Food Agric..

[97] Hashemzadeh F., Rafeie F., Hadipour A., Rezadoust M.H. (2022). Supplementing a phytogenic-rich herbal mixture to heat-stressed lambs: Growth performance, carcass yield, and muscle and liver antioxidant status. Small Rumin. Res..

[98] Hart K.J., Sinclair L.A., Wilkinson R.G., Huntington J.A. (2011). Effect of whole-crop pea (*Pisum sativum* L.) silages differing in condensed tannin content as a substitute for grass silage and soybean meal on the performance, metabolism, and carcass characteristics of lambs. J. Anim. Sci..

[99] Hatami A., Alipour D., Hozhabri F., Tabatabaei M. (2018). Effect of different levels of pomegranate marc with or without polyethylene glycol on performance, nutrients digestibility and protozoal population in growing lambs. Anim. Feed Sci. Technol..

[100] Ibrahim S.L., Hassen A. (2022). Effect of non-encapsulated and encapsulated mimosa (*Acacia mearnsii*) tannins on growth performance, nutrient digestibility, methane and rumen fermentation of South African mutton Merino ram lambs. Anim. Feed Sci. Technol..

[101] Jabalbarezi Hukerdi Y., Fathi Nasri M.H., Rashidi L., Ganjkhanlou M., Emami A. (2020). Supplementing kids diet with olive leaves: Effect on meat quality. Small Rumin. Res..

[102] Jacondino L.R., Poli C.H.E.C., Tontini J.F., Corrêa G.F., Somacal S., Mello R.O., Leal M.L.R., Raimondo R.F.S., Riet-Correa B., Muir J.P. (2022). *Acacia mearnsii* tannin extract and α-tocopherol supplementation in lamb diet: Effects on growth performance, serum lipid peroxidation and meat quality. Anim. Feed Sci. Technol..

[103] Kafle D., Lee J.H., Min B.R., Kouakou B. (2021). Carcass and meat quality of goats supplemented with tannin-rich peanut skin. J. Agric. Food Res..

[104] Kamel H.E.M., Al-Dobaib S.N., Salem A.Z.M., López S., Alaba P.A. (2018). Influence of dietary supplementation with sunflower oil and quebracho tannins on growth performance and meat fatty acid profile of *Awassi lambs*. Anim. Feed Sci. Technol..

[105] Kamel H.E.M., Al-Dobaib S.N., Salem A.Z.M. (2019). Dietary supplementation of sunflower oil and quebracho tannins in sheep feeding: In vivo nutrient digestibility, nitrogen utilization and in vitro ruminal degradation kinetics. J. Sci. Food Agric..

[106] Karamnejad K., Sari M., Salari S., Chaji M. (2019). Effects of nitrogen source on the performance and feeding behavior of lambs fed a high concentrate diet containing pomegranate peel. Small Rumin. Res..

[107] Kazemi M. (2021). An investigation on chemical/mineral compositions, ruminal microbial fermentation, and feeding value of some leaves as alternative forages for finishing goats during the dry season. AMB Exp..

[108] Lee J.H., Min B.R. (2021). Carcass Characteristics and Meat Quality of Kiko Crossbred Male Goats as Influenced by Feeding Phytochemical Tanning Containing Supplementations. Agric. Sci..

[109] Lima P.R., Apdini T., Freire A.S., Santana A.S., Moura L.M.L., Nascimento J.C.S., Rodrigues R.T.S., Dijkstra J., Neto A.F.G., Queiroz M.A.Á. (2019). Dietary supplementation with tannin and soybean oil on intake, digestibility, feeding behavior, ruminal protozoa and methane emission in sheep. Anim. Feed Sci. Technol..

[110] Mahgoub O., Kadim I.T., Tageldin M.H., Al-Marzooqi W.S., Khalaf S.Q., Ali A.A. (2008). Clinical profile of sheep fed non-conventional feeds containing phenols and condensed tannins. Small Rumin. Res..

[111] Majewska M.P., Kowalik B. (2020). Growth Performance, Carcass Characteristics, Fatty Acid Composition, and Blood Biochemical Parameters of Lamb Fed Diet with the Addition of Lingonberry Leaves and Oak Bark. Eur. J. Lipid Sci. Technol..

[112] Manuel-Pablo A., Elghandour M.M.Y., Olivares-Pérez J., Rojas-Hernández S., Cipriano-Salazar M., Cruz-Lagunas B., Camacho-Diaz L.M. (2020). Productive performance, rumen fermentation and carcass yield of goats supplemented with cascalote fruit (*Caesalpinia coriaria* J. Wild.). Agrofor. Sys..

[113] Mavasa N.O., Ng’Ambi J.W., Chitura T. (2022). Partial replacement of maize meal with high-tannin sorghum meal affects finishing and methane emissions of Pedi goats. S. Afr. J. Anim. Sci..

[114] Maxiselly Y., Chiarawipa R., Somnuk K., Hamchara P., Cherdthong A., Suntara C., Prachumchai R., Chanjula P. (2022). Digestibility, Blood Parameters, Rumen Fermentation, Hematology, and Nitrogen Balance of Goats after Receiving Supplemental Coffee Cherry Pulp as a Source of Phytochemical Nutrients. Vet. Sci..

[115] Menci R., Coppa M., Torrent A., Natalello A., Bernardo V., Giuseppe L., Alessandro P., Vincent N. (2021). Effects of two tannin extracts at different doses in interaction with a green or dry forage substrate on in vitro rumen fermentation and biohydrogenation. Anim. Feed Sci. Technol..

[116] Mendo O.H., Ayala Monter M.A., Ortiz S.L., Sánchez D.H., Osorio G.A., Martínez R.M. (2023). Effect of *Guazuma ulmifolia* tannins in the diet of Pelibuey lambs on animal performance and meat characteristics. Emir. J. Food. Agric..

[117] Min B.R., Solaiman S., Gurung N., Behrends J., Eun J.-S., Taha E., Rose J. (2012). Effects of pine bark supplementation on performance, rumen fermentation, and carcass characteristics of Kiko crossbred male goats. J. Anim. Sci..

[118] Min B.R., Solaiman S., Terrill T., Ramsay A., Mueller-Harvey I. (2015). The effects of tannins-containing ground pine bark diet upon nutrient digestion, nitrogen balance, and mineral retention in meat goats. J. Anim. Sci. Biotechnol..

[119] Molina-Alcaide E., Yáñez-Ruiz D.R. (2007). A comparative study of the effect of two-stage olive cake added to alfalfa on digestion and nitrogen losses in sheep and goats. Animal.

[120] Moreira G.D., Lima P.D.M.T., Borges B.O., Primavesi O., Longo C., McManus C., Abdalla A., Louvandini H. (2013). Tropical tanniniferous legumes used as an option to mitigate sheep enteric methane emission. Trop. Anim. Health Prod..

[121] Narjisse H., Elhonsali M.A., Olsen J.D. (1995). Effects of oak (*Quercus ilex*) tannins on digestion and nitrogen balance in sheep and goats. Small Rumin. Res..

[122] Ngámbi J.W., Selapa M.J., Brown D., Manyelo T.G. (2022). The effect of varying levels of purified condensed tannins on performance, blood profile, meat quality and methane emission in male Bapedi sheep fed grass hay and pellet-based diet. Trop. Anim. Health Prod..

[123] Nobre P.T., Munekata P.E.S., Costa R.G., Carvalho F.R., Ribeiro N.L., Queiroga R.C.R.E., Sousa S., da Silva A.C.R., Lorenzo J.M. (2020). The impact of dietary supplementation with guava (*Psidium guajava* L.) agroindustrial waste on growth performance and meat quality of lambs. Meat Sci..

[124] Obeidat B.S., Alrababah M.A., Abdullah A.Y., Alhamad M.N., Gharaibeh M.A., Rababah T.M., Ishmais M.A.A. (2011). Growth performance and carcass characteristics of *Awassi lambs* fed diets containing carob pods (*Ceratonia siliqua* L.). Small Rumin. Res..

[125] Olafadehan O.A., Okunade S.A., Njidda A.A., Kholif A.E., Kolo S.G., Alagbe J.O. (2020). Concentrate replacement with *Daniellia oliveri* foliage in goat diets. Trop. Anim. Health Prod..

[126] Olafadehan O.A. (2011). Changes in haematological and biochemical diagnostic parameters of Red Sokoto goats fed tannin-rich *Pterocarpus erinaceus* forage diets. Vet. Arh..

[127] Orlandi T., Stefanello S., Mezzomo M.P., Pozo C.A., Kozloski G.V. (2020). Impact of a tannin extract on digestibility and net flux of metabolites across splanchnic tissues of sheep. Anim. Feed Sci. Technol..

[128] Osakwe I.I., Drochner W. (2006). Nutritive value of *Morinda lucida* and its fermentation parameters in West African dwarf (WAD) sheep when fed as supplement to grass hay. Small Rumin. Res..

[129] Pannell D., Kouakou B., Terrill T.H., Ogunade I.M., Estrada-Reyes Z.M., Bryant V., Taiwo G., Idowu M., Pech-Cervantes A.A. (2022). Adding dried distillers grains with solubles influences the rumen microbiome of meat goats fed lespedeza or alfalfa-based diets. Small Rumin. Res..

[130] Pathak A.K., Dutta N., Pattanaik A.K., Chaturvedi V.B., Sharma K. (2017). Effect of condensed tannins from *Ficus infectoria* and *Psidium guajava* leaf meal mixture on nutrient metabolism, methane emission and performance of lambs. Asian-Austral. J. Anim. Sci..

[131] Peng K., Shirley D.C., Xu Z., Huang Q., McAllister T.A., Chaves A.V., Acharya S., Liu C., Wang S., Wang Y. (2016). Effect of purple prairie clover (*Dalea purpurea* Vent.) hay and its condensed tannins on growth performance, wool growth, nutrient digestibility, blood metabolites and ruminal fermentation in lambs fed total mixed rations. Anim. Feed Sci. Technol..

[132] Pérez B.I.C., Rojas-Román L.A., Estrada-Angulo A., Muro O.C., Barreras A., Plascencia A. (2021). Effects of long-term supplementation of different levels of tannins extract on meat quality and carcass traits of hairy lambs. Adv. Anim. Vet. Sci..

[133] Pimentel P.R.S., Pellegrini C.B., Lanna D.P.D., Brant L.M.S., Ribeiro C., Silva T.M., Barbosa A.M., da Silva Júnior J.M., Bezerra L.R., Oliveira R.L. (2021). Effects of *Acacia mearnsii* extract as a condensed-tannin source on animal performance, carcass yield and meat quality in goats. Anim. Feed Sci. Technol..

[134] Priolo A., Waghorn G.C., Lanza M., Biondi L., Pennisi P. (2000). Polyethylene glycol as a means for reducing the impact of condensed tannins in carob pulp: Effects on lamb growth performance and meat quality. J. Anim. Sci..

[135] Priolo A., Bella M., Lanza M., Galofaro V., Biondi L., Barbagallo D., Salem HBen Pennisi P. (2005). Carcass and meat quality of lambs fed fresh sulla (*Hedysarum coronarium* L.) with or without polyethylene glycol or concentrate. Small Rumin. Res..

[136] Priolo A., Vasta V., Fasone V., Lanza C.M., Scerra M., Biondi L., Bella M., Whittington F.M. (2009). Meat odour and flavour and indoles concentration in ruminal fluid and adipose tissue of lambs fed green herbage or concentrates with or without tannins. Animal.

[137] Raju J., Sahoo B., Chandrakar A., Garg A.K., Mohanta R.K. (2018). Effect of varied sources of tannin on micro-mineral bioavailability in goats fed oak leaves based diets. Anim. Nutr. Feed Technol..

[138] Reynolds D., Min B.R., Gurung N., McElhenney W., Lee J.H., Solaiman S., Bolden-Tiller O. (2020). Influence of tannin-rich pine bark supplementation in the grain mixes for meat goats: Growth performance, blood metabolites, and carcass characteristics. Anim. Nutr..

[139] Rojas-Román L.A., Castro-Pérez B.I., Estrada-Angulo A., Angulo-Montoya C., Yocupicio-Rocha J.A., López-Soto M.A., Barreras A., Zinn R.A., Plascencia A. (2017). Influence of long-term supplementation of tannins on growth performance, dietary net energy and carcass characteristics: Finishing lambs. Small Rumin. Res..

[140] Salami S.A., Valenti B., O’Grady M.N., Kerry J.P., Mattioli S., Licitra G., Luciano G., Priolo A. (2019). Influence of dietary cardoon meal on growth performance and selected meat quality parameters of lambs, and the antioxidant potential of cardoon extract in ovine muscle homogenates. Meat Sci..

[141] Sánchez N., Mendoza G.D., Martínez J.A., Hernández P.A., Camacho Diaz L.M., Lee-Rangel H.A., Vazquez A., Flores Ramirez R. (2018). Effect of *Caesalpinia coriaria* fruits and soybean oil on finishing lamb performance and meat characteristics. BioMed Res. Int..

[142] Santos S.K., dos Rosset M., Miqueletto M.M., Jesus R.M.M., de Sotomaior C.S., de Macedo R.E.F. (2021). Effects of dietary supplementation with quebracho tannins on oxidation parameters and shelf life of lamb meat. Food Sci. Technol..

[143] Sena J.A.B., Villela S.D.J., Santos R.A., Pereira I.G., Castro G.H.F., Mourthé M.H.F., Bonfá C.S., Martins P.G.M.A. (2015). Intake, digestibility, performance, and carcass traits of rams provided with dehydrated passion fruit (*Passiflora edulis* f. flavicarpa) peel, as a substitute of Tifton 85 (*Cynodon* spp.). Small Rumin. Res..

[144] Seyedin S.M.V., Mojtahedi M., Farhangfar S.H., Ghavipanje N. (2022). Partial substitution of alfalfa hay by Berberis vulgaris leaf modulated the growth performance, meat quality and antioxidant status of fattening lambs. Vet. Med. Sci..

[145] Śliwiński B.J., Kreuzer M., Wettstein H.-R., Machmüller A. (2002). Rumen fermentation and nitrogen balance of lambs fed diets containing plant extracts rich in tannins and saponins, and associated emissions of nitrogen and methane. Arch. Anim. Nutr..

[146] Sinz S., Leparmarai P.T., Liesegang A., Ortmann S., Kreuzer M., Marquardt S. (2021). Effects of dietary grapeseed extract on performance, energy and nitrogen balance as well as methane and nitrogen losses of lambs and goat kids. Br. J. Nutr..

[147] Solaiman S., Thomas J., Dupre Y., Min B.R., Gurung N., Terrill T.H., Haenlein G.F.W. (2010). Effect of feeding sericea lespedeza (*Lespedeza cuneata*) on growth performance, blood metabolites, and carcass characteristics of Kiko crossbred male kids. Small Rumin. Res..

[148] Soltan Y.A., Morsy A.S., Sallam S.M.A., Lucas R.C., Louvandini H., Kreuzer M., Abdalla A.L. (2013). Contribution of condensed tannins and mimosine to the methane mitigation caused by feeding *Leucaena leucocephala*. Arch. Anim. Nutr..

[149] Soltani Nezhad B., Dayani O., Tahmasbi R., Khezri A. (2016). Effects of Replacing Alfalfa Hay and Wheat Straw by Pistachio by-Product Silage and Date Waste on the Performance and Blood Parameters of Fattening Lambs. Iran. J. Appl. Anim. Sci..

[150] Taethaisong N., Paengkoum S., Kaewwongsa W., Onjai-Uea N., Thongpea S., Paengkoum P. (2023). The Effect of Neem Leaf Supplementation on Growth Performance, Rumen Fermentation, and Ruminal Microbial Population in Goats. Animals.

[151] Uushona T., Chikwanha O.C., Katiyatiya C.L.F., Strydom P.E., Mapiye C. (2023). Production and meat quality attributes of lambs fed varying levels of *Acacia mearnsii* leaf-meal as replacement for Triticum aestivum bran. Meat Sci..

[152] Vasta V., Pennisi P., Lanza M., Barbagallo D., Bella M., Priolo A. (2007). Intramuscular fatty acid composition of lambs given a tanniniferous diet with or without polyethylene glycol supplementation. Meat Sci..

[153] Vasta V., Priolo A., Scerra M., Hallett K.G., Wood J.D., Doran O. (2009). Δ9 desaturase protein expression and fatty acid composition of longissimus dorsi muscle in lambs fed green herbage or concentrate with or without added tannins. Meat Sci..

[154] Vasta V., Mele M., Serra A., Scerra M., Luciano G., Lanza M., Priolo A. (2009). Metabolic fate of fatty acids involved in ruminal biohydrogenation in sheep fed concentrate or herbage with or without tannins. J. Anim. Sci..

[155] Wang S., Terranova M., Kreuzer M., Marquardt S., Eggerschwiler L., Schwarm A. (2018). Supplementation of pelleted hazel (*Corylus avellana*) leaves decreases methane and urinary nitrogen emissions by sheep at unchanged forage intake. Sci. Rep..

[156] Wang Z., Guo L., Ding X., Li F., Xu H., Li S., Wang X., Li K., Yue X. (2023). Supplementation of chestnut tannins in diets can improve meat quality and antioxidative capability in Hu lambs. Meat Sci..

[157] Wu P., Fu X., Wang H., Hou M., Shang Z. (2021). Effect of silage diet (sweet sorghum vs. whole-crop corn) and breed on growth performance, carcass traits, and meat quality of lambs. Animals.

[158] Yusuf A.L., Adeyemi K.D., Roselina K., Alimon A.R., Goh Y.M., Samsudin A.A., Sazili A.Q. (2018). Dietary supplementation of different parts of *Andrographis paniculata* affects the fatty acids, lipid oxidation, microbiota, and quality attributes of longissimus muscle in goats. Food Res. Int..

[159] Zhao M.D., Di L.F., Tang Z.Y., Jiang W., Li C.Y. (2019). Effect of tannins and cellulase on growth performance, nutrients digestibility, blood profiles, intestinal morphology and carcass characteristics in Hu sheep. Asian Australas. J. Anim. Sci..

