# Supplementary material for: Meta-Analysis of Dietary Tannins in Small Ruminant Diets: Effects on Growth Performance, Serum Metabolites, Antioxidant Status, Ruminal Fermentation, Meat Quality, and Fatty Acid Profile
Source: Animals (Basel). 2025 Feb 19;15(4):596. doi: 10.3390/ani15040596 (PMC11851383; doi:10.3390/ani15040596)
Supplement: Supplementary file 1 [file animals-15-00596-s001.zip › animals-3366149-supplementary(4).pdf]

Animals – **Supplementary Materials**

Supplementary Table S1. Summary of the studies included in the meta-analysis

Reference	Conc. (g/kg DM)	Type	Species	Country	Initial weight (g/kg DM)	Age (months)	Experimental duration (days)	Administration form	Tannin source	Concentrate in the diet (g/kg DM)
**Abdallah Filho et al., 2016 (1)**	0_40	TT	Sheep	Brazil	7_20	1_6	31_90	Plant	*Orbignya phalerata* and *Combretum leprosum*	400_600
**Abdallah Filho et al., 2017 (2)**	0_40	TT	Sheep	Brazil	7_20	1_6	31_90	Plant	*Orbignya phalerata* and *Combretum leprosum*	400_600
**Abarghuei et al., 2010 (3)**	21_40	TT	Sheep	Iran	61_71	NR	31_90	Plant	*Vitis vinifera*	<400
**Abdullah et al., 2017 (4)**	0_20	TT	Sheep	Egypte	41_60	13_24	5_30	Plant	*Orbignya phalerata* and *Combretum leprosum*	400_600
**Adejoro et al., 2020 (5)**	0_20	CT	Sheep	South Africa	NR	1_6	91_195	Extract	*Acacia mearnsii*	400_600
**Aghamohamadi et al., 2014 (6)**	0_20	TT	Sheep	Iran	41_60	NR	91_195	Plant	*Quercus persica*	400_600
**Al-Dobaib, 2009 (7)**	0_40	CT	Sheep	Argentina	41_60	NR	31_90	Extract	Quebracho	NR
**Animut et al., 2008 (8)**	41_100 and >100	CT	Goat	USA	21_40	7_12	5_30	Plant	*Kobe lespedeza*	NR
**Archimède et al., 2015 (9)**	0_100	CT	Sheep	French	NR	NR	91_195	Plant	*Glyricidia sepium, Leucaena leucocephala, Manihot esculenta*	NR
**Balehegn et al., 2014 (10)**	0_20	TT	Goat	Ethiopia	7_20	7_12	NR	Plant	*Ficus thonningii*	<400
**Ban et al., 2022 (11)**	0_20	CT	Goat	Thailand	21_40	NR	5_30	Plant	*Garcinia mangostana* L.	<400
**Bandeira et al., 2017 (12)**	41_100 and >100	CT	Sheep	Brazil	7_20	1_6	31_90	Plant	*Mimosa tenuiflora*	400_600
**Batchu et al., 2021 (13)**	21_40	CT	Goat	USA	21_40	7_12	31_90	Plant	*Sericea lespedeza*	<400
**Ben Salem et al., 2005 (14)**	0_20	TT	Sheep	Tunisia	-	NR	31_90	Plant	*Acacia cyanophylla*	NR
**Chanjula et al., 2022 (15)**	0_20	CT	Goat	Thailand	21_40	NR	31_90	Plant	Dried Kratom Leaves	>600
**Chikwanha et al., 2019a (16)**	21_100	CT	Sheep	South Africa	21_40	1_6	31_90	Plant	*Vitis vinifera*	NR

**Chikwanha et al., 2019b (17)**	21_100	TT	Sheep	South Africa	41_60	1_6	31_90	Plant	*Vitis vinifera*	NR
**Costa et al., 2021 (18)**	0_100	CT	Sheep	Brazil	21_40	NR	31_90	Extract	*Acacia mearnsii*	400_600
**Dawson et la., 1999 (19)**	41_100	CT	Sheep	UK	21_40	NR	31_90	Extract	Quebracho	NR
**Dentinho et al., 2014 (20)**	0_40	CT	Sheep	Portugal	41_60	25_48	31_90	Extract	*Citrus ladanifer*	400_600
**Dey et al., 2008 (21)**	0_20	CT	Sheep	India	7_20	1_6	91_195	Plant	*Ficus infectoria*	>600
**El-Meccawi et al., 2008 (22)**	>100	CT	Sheep	Israel	21-60	7_12	5_30	Plant	*Acacia saligna*	NR
**Emami et al., 2015 (23)**	0_20	TT	Goat	Iran	7_20	NR	31_90	Plant	Pomegranate seed pulp	>600
**Emami et al., 2015 (24)**	0_20	TT	Goat	Iran	7_20	1_6	31_90	Plant	Pomegranate seed pulp	>600
**Emami et al., 2015 (25)**	0_20	CT	Goat	Iran	7_20	1_6	31_90	Plant	Pomegranate seed pulp	>600
**Fernandez et al., 2021 (26)**	0_40	TT	Sheep	Brazil	21_40	NR	31_90	Plant	*Mimosa tenuiflora* hay	400_600
**Froutan et al., 2015 (27)**	0_20	CT	Goat	Iran	7_20	1_12	91_195	Plant	Oak acorn	>600
**Galvão et al., 2020 (28)**	0_20	TT	Goat	Brazil	21_40	1_6	5_30	Plant	Tamarind residue	400_600
**García-Salas et al., 2022 (29)**	0_20	CT	Sheep	Mexico	21_40	NR	31_90	Extract	*Acacia mearnsii*	NR
**Guerreiro et al., 2019 (30)**	0_40	CT	Sheep	Portugal	7_20	1_6	31_90	Plant and extract	*Cistus ladanifer*	<400
**Ghaffari et al., 2013 (31)**	0_40	TT	Sheep	Iran	41_60	NR	31_90	Plant	Pistachio by-products	400_600
**Giller et al., 2021 (32)**	21_40	TT	Sheep	Switzerland	NR	1_6	31_90	Plant	Grape seed	400_600
**Girard et al., 2014 (33)**	21_40 and >100	TT	Sheep	Switzerland	21_40	1_6	91_195	Plant	*Lotus corniculatus* L.	<400
**Hashemzadeh et al., 2022 (34)**	0_20	TT	Sheep	Iran	41_60	7_12	5_90	Plant	Herbal mixture	>600
**Hart et al., 2011 (35)**	21_100	TT	Sheep	UK	21_40	NR	91_195	Plant	Pea silage	400_600
**Hatami et al., 2017 (36)**	0_40	TT	Sheep	Iran	21_40	1_6	31_90	Plant	Pomegranate marc	>600
**Ibrahim et Hassen, 2022 (37)**	0_20	TT	Sheep	South Africa	21_40	NR	91_195	Extract	Mimosa	400_600
**Jabalbarezi Hukerdi et al., 2019 (38)**	0_20	TT	Goat	Iran	21_40	1_6	31_90	Plant	Olive leaf	>600
**Jacondino et al., 2022 (39)**	41_100	TT	Sheep	Brazil	21_40	1_6	31_90	Extract	*Acacia mearnsii*	<400

**Kafle et el., 2021 (40)**	21_100 and >100	CT	Goat	USA	41_60	7_12	31_90	Plant	Peanut skin	NR
**Kamel et al., 2018 (41)**	0_40	CT	Sheep	Saudi Arabia	21_40	1_6	31_90	Extract	Quebracho	>600
**Kamel et al., 2019 (42)**	0_40	CT	Sheep	Saudi Arabia	41_60	13_24	31_90	Extract	Quebracho	>600
**Karamnejad et al., 2019 (43)**	0_20	TT	Sheep	Iran	7_20	1_6	91_195	Plant	Pomegranate pulp	>600
**Kazemi et al., 2021 (44)**	0_20	TT	Goat	Iran	7_20	7_12	31_90	Plant	*Cydonia oblonga Mill.),Pyrus communis L., Olea europaea L., Prunus domestica L., Prunus domestica L., Prunus cerasus L.,* and *Diospyros kaki L.*	400_600
**Lee et Min, 2021 (45)**	21_100	CT	Goat	USA	21_40	7_12	31_90	Plant	Bermuda grass and *Sericea lespedeza*	>600
**Lima et al., 2019 (46)**	21_40	TT	Sheep	Brazil	NR	NR	31_90	Extract	*Acacia mearnsii*	400_600
**Mahgoub et al., 2008 (47)**	0_20	CT	Sheep	Omâ	21_40	7_12	91_195	Plant	B-products	NR
**Majewska and Kowalik, 2019 (48)**	0_20	TT	Sheep	Poland	7_20	NR	31_90	Plant	Lingonberry leaves and Oak bark	>600
**Manuel-Pablo et al., 2020 (49)**	0_100	TT	Goat	Mexico	7_20	13_24	31_90	Extract	*Caesalpinia coriaria* (Jacq.) Willd	NR
**Mavasa et al., 2022 (50)**	0_20	TT	Goat	South Africa	7_20	7_12	5_30	Plant	Sorghum	NR
**Maxiselly et al., 2022 (51)**	0_20	TT	Goat	Thailand	21_40	7_12	31_90	Plant	Coffee Cherry Pulp	>600
**Menci et al., 2023 (52)**	0_20	TT	Sheep	France	21_40	1_6	5_30	Plant	Chestnut shells and Sainfoin	<400
**Mendo et al., 2023 (53)**	0_40	TT	Sheep	Mexico	21_40	1_6	31_90	Plant	*Guazuma ulmifolia*	>600
**Min et al., 2012 (54)**	0_40	CT	Goat	USA	21_40	1_6	31_90	Plant	Pine bark	>600
**Min et al. 2015 (55)**	0_40	CT	Goat	USA	21_40	1_6	5_30	Plant	Pine bark	NR

**Molina-Alcaide and Yanez-Ruiz, 2007 (56)**	0_20	CT	Goat and sheep	Spain	41_60	NR	5_30	Plant	Olive cake	<400
**Moreira et al., 2013 (57)**	21_40	CT	Sheep	Brazil	21_40	7_12	5_30	Plant	*Leucaena leucocephala, Styzolobium aterrimun, Mimosa aesalpiniaefolia*	<400
**Narjisse et al., 1995 (58)**	0_20	TT	Sheep	Morocco	7_40	13_24	5_30	Extract	*Quercus ilex*	NR
**Ngámbi et al., 2022 (59)**	21_100	CT	Sheep	South Africa	21_40	NR	5_30	Extract	-	NR
**Nobre et al., 2020 (60)**	0_20	TT	Sheep	Brazil	21_40	1_6	31_90	Plant	Guava	400_600
**Obeidat et al., 2011 (61)**	0_20	CT	Sheep	Jordan	7_20	1_6	31_90	Plant	*Ceratonia siliqua*	>600
**Olafadehan et al., 2020 (62)**	0_40	CT	Goat	Nigeria	7_20	1_6	31_90	Plant	*Daniellia oliveri*	NR
**Olafadehan, 2011 (63)**	0_100	CT	Goat	Nigeria	7_20	1_6	31_90	Plant	*Pterocarpus erinaceus*	NR
**Orlandi et al., 2020 (64)**	0_20	TT	Sheep	Brazil	41_60	NR	31_90	Extract	*Acacia mearnsii*	NR
**Osakwe and Drochner, 2006 (65)**	0_20	CT	Sheep	Nigeria	21_40	13_24	5_30	Plant	*Morinda lucida*	NR
**Pannell et al., 2022 (66)**	0_20	CT	Goat	USA	21_40	NR	31_90	Plant	Dried distillers grains with solubles	400_600
**Pathak et al., 2017 (67)**	0_20	CT	Sheep	India	7_20	1_6	91_195	Plant	*Ficus sinfectoria* + *Psidium guajava*	NR
**Peng et al., 2016 (68)**	41_100	CT	Sheep	Canada	21_40	1_6	31_90	Plant	*Dalea purpurea*	400_600
**Pérez et al., 2021 (69)**	0_20	TT	Sheep	Mexico	21_40	1_6	31_90	Extract	-	NR
**Pimentel et al., 2021 (70)**	0_100	CT	Goat	Brazil	7_20	1_6	31_90	Extract	*Acacia mearnsii*	400_600
**Priolo et al., 2000 (71)**	21_40	CT	Sheep	Italy	7_20	1_6	91_195	Plant	*Ceratonia siliqua*	400_600
**Priolo et al., 2005 (72)**	0_20	TT	Sheep	Italy	21_40	1_6	31_90	Plant	*Hedysarum coronarium*	>600
**Priolo et al., 2009 (73)**	21_40	TT	Sheep	Italy	21_40	1_6	91_195	Plant	*Schinopsis lorentzii*	>600
**Raju et al., 2018 (74)**	21_100	TT	Goat	India	7_20	7_12	91_195	Plant	Oak Leaves	<400
**Reynolds et al. 2020 (75)**	21_40	CT	Goat	USA	21_40	7_12	31_90	Plant	Pine bark	NR
**Roja-Romàn et al., 2017 (76)**	0_20	TT	Sheep	México	21_40	NR	5_30	Extract	-	>600
**Salami et al., 2018 (77)**	21_40	HT	Sheep	Italy	7_20	NR	31_90	Extract	Chestnut (HT) and Tannins T80® (CT)	>600

**Sánchez et al., 2018 (78)**	0_20	CT	Sheep	Mexico	21_40	NR	31_90	Plant	*Caesalpinia coriaria*	NR
**Santos et al., 2020 (79)**	0_100	TT	Sheep	Brazil	7_20	1_6	91_195	Extract	Quebracho	<400
**Sena et al., 2015 (80)**	0_20	TT	Sheep	Brazil	21_40	NR	5_90	Plant	*Passiflora edulis f. flavicarpa*	400_600
**Seyedin et al., 2022 (81)**	0_20	TT	Sheep	Iran	21_40	1_6	31_90	Plant	*Berberis vulgaris*	>600
**Sliwinski et al., 2002 (82)**	0_20	TT	Sheep	Switzerland	21_40	1_6	91_195	Extract	-	400_600
**Sinz et al., 2020 (83)**	21_40	TT	Sheep and goat	Mexico	7_20	1_6	31_90	Plant	Grapeseed extract	400_600 and >600
**Solaiman et al., 2010 (84)**	0_40	CT	Goat	USA	21_40	1_6	31_90	Plant	*Servicea lespedeza*	NR
**Soltan et al., 2013 (85)**	0_20	TT	Sheep	Brazil	61_71	1_6	31_90	Plant	*Leucaena leucocephala*	<400
**Soltani et al., 2016 (86)**	0_20	TT	Sheep	Iran	21_40	1_6	31_90	Plant	Pistachio by-products silage	400_600
**Taethaisong et al., 2023 (87)**	0_20	CT	Goat	Thailand	7_20	NR	31_90	Plant	Neem Leaf	NR
**Uushona et al., 2023 (88)**	0_40	CT	Sheep	South Africa	21_40	1_6	31_90	Plant	*Acacia mearnsii* leaf	NR
**Vasta et al., 2007 (89)**	21_40	CT	Sheep	Italy	NR	1_6	31_90	Plant	*Ceratonia siliqua*	400_600
**Vasta et al., 2009 (90)**	21_40	TT	Sheep	Italy	21_40	1_6	31_90	Plant	*Vicia sativa*	>600
**Vasta et al., 2009 (91)**	41_100	TT	Sheep	Italy	21_40	1_6	31_90	Plant	*schinopsis lorentzii*	>600
**Wang et al., 2018 (92)**	0_20	TT	Sheep	France	61_71	13_24	31_90	Plant	*Corylus avellana*	>600
**Wang et al., 2023 (93)**	0_20	TT	Sheep	China	21_40	1_6	31_90	Plant	Chestnut	>600
**Wu et al., 2021 (94)**	0_20	CT	Sheep	China	21_40	1_6	31_90	Plant	Sweet sorghum silage	<400
**Yisehak et al., 2016 (95)**	41_100	CT	Goat	Ethiopia	7_40	13_24	31_90	Plant	*Albizia schimperiana* and *Ficus elastica*	NR
**Yusuf et al., 2018 (96)**	0_20	TT	Goat	Malaysia	7_20	1_6	91_195	Plant	*Andrographis paniculata* leaves	400_600
**Zhao et al., 2019 (97)**	0_20	TT	Sheep	China	21_40	1_6	31_90	Extract	-	400_600

TT: total tannins; CT: condensed tannins; HT: hydrolyzable tannins; UK: United Kingdom; USA: United States of America; NR: not reported.

Supplementary Table S2. Meta-regression of the covariate effect on weighted mean differences (WMD) between tannin and control treatments on diet nutrient intake and digestibility, fermentation, blood parameters, carcass characteristics, meat quality, and fatty acid profile. The adjusted R^2 is the proportion of between-study variance (heterogeneity) explained by the covariate.

Dependent variable (Y, WMD)[2]	Intercept	Continent	Species	Animals age (months)	Initial weight (kg)	Exp. duration (days)	Tannins CC (g/kg DM)	Tannin type	Admin. form	Conc. Diet	Adjusted R^2 (%)
**Intake (g/day)**											
**Dry matter**	-101.34 (0.14)	21.12 (0.80)	188.96 (0.01)	1.32 (0.01)	0.93 (0.20)	60.45 (0.72)	-2.22 (0.98)	13.0 (0.52)	9.58 (0.65)	11.27 (0.18)	4.54
**Crude protein**	-0.04 (<0.001)	5.07 (0.08)	4.05 (0.06)	1.58 (<0.001)	0.50 (0.85)	2.11 (0.80)	-1.66 (0.02)	2.39 (0.40)	-0.23 (0.20)	6.39 (<0.001)	91.59
**NDF**	22.50 (<0.001)	27.17 (0.24)	-	0.63 (<0.001)	0.26 (0.66)	13.23 (0.52)	-1.56 (0.008)	13.44 (0.24)	0.19 (0.45)	24.12 (0.20)	52.31
**Digestibility (g/kg DM)**											
**DM**	4.35 (<0.001)	3.19 (0.11)	0.48 (0.43)	-0.84 (0.002)	-1.31 (0.23)	1.43 (0.03)	-9.24 (<0.001)	0.17 (0.43)	-0.81 (0.74)	0.70 (0.73)	68.91
**CP**	3.95 (<0.001)	7.35 (0.07)	2.66 (0.29)	-1.56 (<0.001)	-1.40 (0.59)	2.40 (0.29)	-0.41 (<0.001)	2.71 (0.16)	-1.42 (0.30)	4.96 (0.17)	77.24
**NDF**	2.86 (0.03)	0.56 (0.11)	-0.46 (0.55)	-1.51 (0.25)	-0.83 (0.36)	-0.43 (0.27)	-0.55 (0.02)	-0.23 (0.17)	-0.11 (0.51)	-0.02 (0.22)	17.62
**ADF**	12.10 (<0.001)	2.85 (0.17)	0.32 (0.68)	-0.60 (0.10)	-1.23 (0.65)	1.70 (0.05)	-1.21 (0.001)	0.68 (0.14)	-0.54 (0.76)	1.69 (0.20)	72.23
**Fermentation parameters**											
**Rumen pH**	-0.45 (<0.001)	0.12 (0.92)	0.57 (0.06)	0.003 (0.02)	0.54 (0.08)	0.21 (0.07)	0.84 (0.67)	0.69 (<0.001)	0.04 (0.46)	0.42 (0.78)	67.12
**NH_3-N (mg/dL)**	-1.94 (0.003)	-0.81 (0.42)	-0.004 (0.001)	-0.58 (<0.001)	-0.54 (0.04)	-0.39 (0.23)	0.32 (0.75)	-0.63 (0.48)	-0.50 (0.86)	-0.69 (0.21)	44.81
**SCFA, mol/100 mol**											
**Acetate**	0.34 (0.004)	-0.16 (<0.001)	-0.86 (0.06)	-0.07 (0.06)	-0.41 (0.56)	-0.21 (0.73)	-0.07 (0.70)	0.05 (0.02)	0.16 (0.10)	-0.16 (0.68)	55.04

**Propionate**	1.99 (0.02)	0.64 (0.10)	1.81 (0.02)	0.40 (0.02)	1.56 (0.38)	0.66 (0.61)	-0.55 (0.52)	0.49 (0.88)	0.29 (0.63)	0.33 (0.01)	53.35
**TVFA**	-2.71 (0.06)	0.98 (0.07)	0.50 (0.15)	0.16 (0.32)	0.19 (0.62)	0.14 (0.04)	0.22 (0.56)	0.04 (0.55)	-0.06 (0.61)	0.94 (0.02)	46.79
**N metabolism**											
**N intake**	-1.80 (<0.001)	3.79 (0.08)	1.10 (0.94)	0.80 (0.29)	0.98 (0.21)	0.11 (0.04)	2.08 (0.89)	0.81 (0.55)	-0.69 (0.02)	1.02 (0.32)	65.79
**N urine**	-0.71 (<0.001)	-1.57 (0.65)	-	-0.62 (0.12)	-2.42 (<0.001)	-0.73 (0.85)	-2.67 (0.25)	-1.07 (0.64)	-1.50 (0.30)	-0.89 (0.98)	59.77
**N fecal**	11.68 (0.09)	-1.32 (0.09)	0.86 (0.96)	2.24 (0.04)	1.32 (0.15)	0.89 (0.40)	5.80 (0.20)	1.16 (0.67)	0.63 (0.99)	1.07 (0.11)	22.71
**N retained**	-6.21 (0.04)	3.91 (0.09)	1.78 (0.53)	-0.39 (0.09)	-0.37 (0.18)	0.25 (0.02)	1.32 (0.94)	1.21 (0.75)	-0.22 (0.29)	1.17 (0.63)	31.84
**Blood metabolites**											
**Blood urea nitrogen**	-0.58 (<0.001)	-1.79 (0.12)	-1.41 (0.34)	-1.49 (0.64)	-1.28 (0.68)	-1.56 (0.07)	0.39 (0.26)	-1.41 (0.39)	-0.69 (0.49)	-1.13 (0.11)	38.07
**Antioxidant activity**											
**ALT**	0.20 (0.005)	0.30 (0.04)	-	-0.01 (0.31)	-0.01 (0.04)	-0.31 (0.62)	-0.41 (0.20)	-0.08 (0.32)	0.84 (0.05)	0.55 (<0.001)	48.84
**Growth performance**											
**Final body weight (kg)**	0.43 (0.03)	0.01 (0.02)	0.18 (0.20)	0.27 (0.43)	0.62 (0.39)	0.06 (0.58)	-0.70 (0.001)	0.13 (0.20)	-0.08 (0.63)	0.24 (0.38)	13.88
**Average daily gain (g/day)**	-111.64 (<0.001)	0.42 (0.11)	1.94 (0.68)	153.41 (0.07)	391.8 (0.01)	0.08 (0.98)	-2.35 (0.27)	48.66 (0.43)	168.67 (0.006)	167.07 (0.04)	22.14
**Feed conversion ratio**	-0.08 (<0.001)	-1.77 (0.02)	-1.13 (0.02)	-0.41 (<0.001)	-1.69 (0.03)	-0.15 (<0.001)	-1.85 (0.59)	-0.19 (0.46)	-0.22 (0.74)	-1.66 (0.001)	59.20
**Carcass characteristics**											
**Cold carcass weight**	-9.65 (<0.001)	-0.63 (0.06)	-0.19 (0.42)	-0.41 (0.29)	-2.11 (0.34)	-	-0.67 (0.08)	-0.37 (0.90)	-1.06 (0.01)	-0.70 (0.07)	71.43
**Hot carcass weight**	0.12 (0.04)	0.30 (0.02)	-0.19 (0.71)	0.10 (0.15)	-0.44 (0.55)	0.31 (0.01)	-1.80 (0.004))	-0.14 (0.87)	-0.22 (0.71)	-0.05 (0.04)	15.74
**Meat quality**											

**Cooking loss**	-17.97 (0.05)	-0.04 (0.71)	-0.54 (0.21)	-0.21 (0.66)	-4.88 (0.05)	-	0.51 (0.21)	-0.01 (0.29)	-0.55 (0.22)	0.05 (0.75)	21.87
**pH meat**	-0.45 (0.11)	0.07 (0.04)	-0.08 (0.05)	0.92 (0.67)	-0.56 (0.12)	0.13 (0.99)	0.58 (0.57)	0.43 (0.49)	0.37 (0.58)	0.09 (0.06)	12.61
**Fatty acid profile (g/100 g FA)**											
**Stearic (C18:0)**	1.43 (0.01)	-0.70 (0.26)	-0.07 (0.26)	0.003 (0.47)	-0.69 (0.03)	-0.29 (0.25)	-1.01 (0.41)	-0.14 (0.14)	0.25 (0.05)	-0.01 (0.07)	42.33
**Oleic (C18:1 c9)**	-7.73 (0.02)	-0.16 (0.16)	-0.72 (0.29)	2.34 (0.03)	-0.48 (0.61)	0.19 (0.93)	-0.14 (0.44)	-0.86 (0.04)	-1.53 (0.04)	0.31 (0.20)	37.17
**Linoleic (LA, C18:2 ω6)**	-1.61(0.09)	0.79 (0.85)	0.87 (0.76)	0.65 (0.92)	0.96 (0.83)	0.72 (0.37)	1.32 (0.07)	0.72 (0.73)	0.95 (0.57)	0.19 (0.48)	24.92
**Conjugated linoleic acid (CLA, C18:2 c9t11)**	-1.72 (0.34)	-0.14 (0.09)	0.40 (0.82)	0.33 (0.88)	0.71 (0.62)	-	-0.36 (0.93)	-0.14 (0.08)	0.37 (0.85)	-0.14 (0.05)	10.24
**α-Linolenic (ALA, C18:3 ω3)**	-0.30 (0.09)	1.11 (0.71)	-	1.01 (0.99)	1.39 (0.85)	-	-0.20 (0.01)	1.38 (0.27)	1.83 (0.13)	0.09 (0.35)	26.51
**Arachidonic (ARA, C20:4 ω6)**	-1.54 (0.23)	0.03 (0.41)	0.30 (0.45)	0.50 (0.54)	0.90 (0.71)	-	1.64 (0.04)	0.38 (0.34)	0.63 (0.99)	-0.15 (0.50)	18.81
**Eicosapentaenoic (EPA, C20:5 ω3)**	0.48 (0.22)	-0.20 (0.11)	-0.04 (0.07)	0.44 (0.22)	0.14 (0.44)	-	1.70 (0.26)	0.21 (0.09)	0.50 (0.92)	-0.15 (0.24)	19.43
**Doccosapentaenoic (DPA, C22:5 ω3)**	-1.81 (0.004)	-0.02 (0.8)	0.52 (0.66)	0.26 (0.11)	0.40 (0.71)	0.54 (0.04)	1.50 (0.17)	0.56 (0.59)	0.79 (0.74)	0.00 (0.55)	56.69
**Docosahexaenoic (DHA, C22:6 ω3)**	1.90 (0.001)	-	0.21 (0.86)	-	-0.10 (0.87)	-	2.32 (0.02)	-0.13 (0.28)	-0.30 (0.16)	1.14 (0.001)	72.12
**Saturated fatty acids (SFA)**	4.67 (<0.001)	-0.57 (0.10)	0.22 (0.21)	-0.07 (0.82)	0.11 (0.13)	-0.04 (0.01)	-1.76 (0.03)	0.23 (0.03)	0.51 (0.01)	-0.57 (0.08)	61.17
**Monounsaturated fatty acids (MUFA)**	1.26 (0.08)	0.38 (0.36)	-0.56 (0.51)	-0.54 (0.49)	-0.46 (0.28)	-0.34 (0.97)	0.01 (0.08)	-0.41 (0.74)	-0.80 (0.10)	0.38 (0.21)	27.02
**Polyunsaturated fatty acids (PUFA)**	2.84 (0.002)	0.12 (0.15)	0.99 (0.93)	1.03 (0.35)	1.94 (0.26)	0.90 (0.02)	1.60 (0.10)	0.84 (0.50)	0.91 (0.85)	0.12 (0.42)	54.69

Exp. Experimental; CC: Concentration:; Admin.: Administration: Conc.: Concentrate; DM: Dry matter: CP: Crude protein; NDF: Neutral detergent fiber; ADF: Acid detergent fiber; SCFA: Short-chain fatty acids; TVFA: Total volatile fatty acids; ALT: Alanine aminotransferase; SCFA: Short-chain fatty acids; SFA: Saturated fatty acid; MUFA: Monounsaturated fatty acid; PUFA: Polyunsaturated fatty acids.

References

1. Abdalla Filho AL, Dineshkumar D, Barreal M, McManus C, Vasconcelos VR, Abdalla AL, Louvandini H. Performance, metabolic variables and enteric methane production of Santa Inês hair lambs fed Orbignya phalerata and Combretum leprosum. *J. Anim. Physiol. Anim. Nutr.* (Berl) (2017) 101:457–465.

2. Abdalla Filho AL, Corrêa PS, Lemos LN, Dineshkumar D, Issakowicz J, Ieda EH, Lima PMT, Barreal M, McManus C, Mui TS. Diets based on plants from Brazilian Caatinga altering ruminal parameters, microbial community and meat fatty acids of Santa Inês lambs. *Small Rumin. Res.* (2017) 154:70–77.

3. Abarghuei MJ, Rouzbehan Y, Alipour D. The influence of the grape pomace on the ruminal parameters of sheep. *Livest. Sci.* (2010) 132:73–79.

4. Abdullah MAM, Farghaly MM, Youssef IMI. Effect of feeding Acacia nilotica pods to sheep on nutrient digestibility, nitrogen balance, ruminal protozoa and rumen enzymes activity. *J. Anim. Physiol. Anim. Nutr.* (2018) 102:662–669.

5. Adejoro FA, Hassen A, Akanmu AM, Morgavi DP. Replacing urea with nitrate as a non-protein nitrogen source increases lambs' growth and reduces methane production, whereas acacia tannin has no effect. *Anim. Feed Sci. Technol.* (2020) 259:114360.

6. Aghamohamadi N, Hozhabri F, Alipour D. Effect of oak acorn (Quercus persica) on ruminal fermentation of sheep. *Small Rumin. Res.*(2014) 120:42–50.

7. Al Dobaib SN. Effect of different levels of quebracho tannin on nitrogen utilization and growth performance of Najdi sheep fed alfalfa (Medicago sativa) hay as a sole diet. *Anim. Sci. J.* (2009) 80:532–541.

8. Animut G, Puchala R, Goetsch AL, Patra AK, Sahlu T, Varel VH, Wells J. Methane emission by goats consuming different sources of condensed tannins. *Anim. Feed Sci. Technol.* (2008) 144:228–241.

9. Archimède H, Rira M, Barde DJ, Labirin F, Marie-Magdeleine C, Calif B, Périacarpin F, Fleury J, Rochette2 Y, Morgavi DP. Potential of tannin-rich plants, Leucaena leucocephala, Glyricidia sepium and Manihot esculenta, to reduce enteric methane emissions in sheep. *J. Anim. Physiol. Anim. Nutr.* (2016) 100:1149–1158.

10. Balehegn M, Eik LO, Tesfay Y. Replacing commercial concentrate by Ficus thonningii improved productivity of goats in Ethiopia. *Trop. Anim. Health Prod.* (2014) 46:889–894. .

11. Ban C, Paengkoum S, Yang S, Tian X, Thongpea S, Purba RAP, Paengkoum P. Feeding meat goats mangosteen (Garcinia mangostana L.) peel rich in condensed tannins, flavonoids, and cinnamic acid improves growth performance and plasma antioxidant activity under tropical conditions. *J. Appl. Anim. Res.* (2022) 50:307–315.

12. Bandeira PAV, Filho JMP, de Azevêdo Silva AM, Cezar MF, Bakke OA, Silva UL, Borburema JB, Bezerra LR. Performance and carcass characteristics of lambs fed diets with increasing levels of Mimosa tenuiflora (Willd.) hay replacing Buffel grass hay. *Trop. Anim. Health Prod.* (2017) 49:1001–1007.

13. Batchu P, Hazard T, Lee JH, Terrill TH, Kouakou B, Kannan G. High-Condensed Tannin Diet and Transportation Stress in Goats: Effects on Physiological Responses, Gut Microbial Counts and Meat Quality. *Animals* (2021) 11.

14. Salem H Ben, Makkar HPS, Nefzaoui A, Hassayoun L, Abidi S. Benefit from the association of small amounts of tannin-rich shrub foliage (Acacia cyanophylla Lindl.) with soya bean meal given as supplements to Barbarine sheep fed on oaten hay. *Anim. Feed Sci. Technol.* (2005) 122:173–186.

15. Chanjula P, Wungsintaweekul J, Chiarawipa R, Rugkong A, Khonkhaeng B, Suntara C, Cherdthong A. Effect of Feed Supplement Containing Dried Kratom Leaves on Apparent Digestibility, Rumen Fermentation, Serum Antioxidants, Hematology, and Nitrogen Balance in Goats. *Fermentation* (2022) 8.

16. Chikwanha OC, Muchenje V, Nolte JE, Dugan MER, Mapiye C. Grape pomace (Vitis vinifera L. cv. Pinotage) supplementation in lamb diets: Effects on growth performance, carcass and meat quality. *Meat Sci.* (2019) 147:6–12.
17. Chikwanha OC, Raffrenato E, Muchenje V, Nolte J van E, Mapiye C. Effect of grape (Vitis vinifera L. cv. Pinotage) pomace supplementation on nutrient utilization in finisher lambs. *Small Rumin. Res.* (2019) 179:48–55.
18. Costa EI de S, Ribeiro CVDM, Silva TM, Batista ASM, Vieira JF, Barbosa AM, da Silva Júnior JM, Bezerra LR, Pereira ES, Oliveira RL. Effect of dietary condensed tannins inclusion from Acacia mearnsii extract on the growth performance, carcass traits and meat quality of lambs. *Livest. Sci.* (2021) 253:104717.
19. Dawson JM, Buttery PJ, Jenkins D, Wood CD, Gill M. Effects of dietary quebracho tannin on nutrient utilisation and tissue metabolism in sheep and rats. *J. Sci. Food Agric.* (1999) 79:1423–1430.
20. Dentinho MTP, Belo AT, Bessa RJB. Digestion, ruminal fermentation and microbial nitrogen supply in sheep fed soybean meal treated with Cistus ladanifer L. tannins. *Small Rumin. Res.* (2014) 119:57–64.
21. Dey A, Dutta N, Sharma K, Pattanaik AK. Effect of dietary inclusion of Ficus infectoria leaves as a protectant of proteins on the performance of lambs. *Small Rumin. Res.* (2008) 75:105–114.
22. El-Meccawi S, Kam M, Brosh A, Degen AA. Heat production and energy balance of sheep and goats fed sole diets of Acacia saligna and Medicago sativa. *Small Rumin. Res.* (2008) 75:199–203.
23. Emami A, Fathi Nasri MH, Ganjkhanlou M, Rashidi L, Zali A. Dietary pomegranate seed pulp increases conjugated-linoleic and -linolenic acids in muscle and adipose tissues of kid. *Anim. Feed Sci. Technol.* (2015) 209:79–89.
24. Emami A, Nasri MHF, Ganjkhanlou M, Zali A, Rashidi L. Effects of dietary pomegranate seed pulp on oxidative stability of kid meat. *Meat Sci* (2015) 104:14–19. doi: 10.1016/j.meatsci.2015.01.016
25. Emami A, Ganjkhanlou M, Fathi Nasri MH, Zali A, Rashidi L. Pomegranate seed pulp as a novel replacement of dietary cereal grains for kids. *Small Rumin. Res.* (2015) 123:238–245.
26. Fernandes J, Pereira Filho J, Menezes D, Caldas AC, Cavalcante I, Oliveira J, Oliveira R, Júnior JS, Cézar M, Bezerra L. Carcass and meat quality in lambs receiving natural tannins from Mimosa tenuiflora hay. *Small Rumin. Res.* (2021) 198:106362.
27. Froutan E, Azizi O, Sadeghi G, Fatehi F, Lashkari S. Effects of different concentrations of ground oak acorn on growth performance, blood parameters and carcass characteristics of goat kids. *Anim. Prod. Sci.* (2014) 55:87–92.
28. Galvão JM, Silva TM, Silva WP, Pimentel PRS, Barbosa AM, Nascimento TVC, Lima AGVO, Bezerra LR, Oliveira RL. Intake, digestibility, ingestive behavior, and nitrogen balance of goats fed with diets containing residue from tamarind fruit. *Trop. Anim. Health Prod.* (2020) 52:257–264.
29. García-Salas A, Bárcena-Gama JR, Hernández-Sánchez D, Cobos-Peralta MA, González-Muñoz SS, Vaquera-Huerta H, Arias-Margarito L. Fattening performance and carcass characteristics of lambs supplemented with condensed tannins from Acacia mearnsii extract. *S. Afr. J. Anim. Sci.* (2022) 52:498–505.
30. Guerreiro O, Alves SP, Soldado D, Cachucho L, Almeida JM, Francisco A, Santos-Silva J, Bessa RJB, Jerónimo E. Inclusion of the aerial part and condensed tannin extract from Cistus ladanifer L. in lamb diets – Effects on growth performance, carcass and meat quality and fatty acid composition of intramuscular and subcutaneous fat. *Meat Sci.* (2020) 160:107945.
31. Ghaffari MH, Tahmasbi A-M, Khorvash M, Naserian A-A, Ghaffari AH, Valizadeh H. Effects of pistachio by-products in replacement of alfalfa hay on populations of rumen bacteria involved in biohydrogenation and fermentative parameters in the rumen of sheep. *J. Anim. Physiol. Anim. Nutr.* (2014) 98:578–586.

32. Giller K, Sinz S, Messadene-Chelali J, Marquardt S. Maternal and direct dietary polyphenol supplementation affect growth, carcass and meat quality of sheep and goats. *Animal* (2021) 15:100333.
33. Girard M, Dohme-Meier F, Silacci P, Ampuero Kragten S, Kreuzer M, Bee G. Forage legumes rich in condensed tannins may increase n-3 fatty acid levels and sensory quality of lamb meat. *J. Sci. Food Agric.* (2016) 96:1923–1933.
34. Hashemzadeh F, Rafeie F, Hadipour A, Rezadoust MH. Supplementing a phytogenic-rich herbal mixture to heat-stressed lambs: Growth performance, carcass yield, and muscle and liver antioxidant status. *Small Rumin. Res.* (2022) 206:106596.
35. Hart KJ, Sinclair LA, Wilkinson RG, Huntington JA. Effect of whole-crop pea (Pisum sativum L.) silages differing in condensed tannin content as a substitute for grass silage and soybean meal on the performance, metabolism, and carcass characteristics of lambs. *J. Anim. Sci.* (2011) 89:3663–3676.
36. Hatami A, Alipour D, Hozhabri F, Tabatabaei M. Effect of different levels of pomegranate marc with or without polyethylene glycol on performance, nutrients digestibility and protozoal population in growing lambs. *Anim. Feed Sci. Technol.* (2018) 235:15–22.
37. Ibrahim, S. L., & Hassen, A. (2022). Effect of non-encapsulated and encapsulated mimosa (Acacia mearnsii) tannins on growth performance, nutrient digestibility, methane and rumen fermentation of South African mutton Merino ram lambs. *Anim. Feed Sci. Technol.*, 294, 115502.
38. Jabalbarezi Hukerdi Y, Fathi Nasri MH, Rashidi L, Ganjkhanlou M, Emami A. Supplementing kids diet with olive leaves: Effect on meat quality. *Small Rumin. Res.* (2020) 193:106258.
39. Jacondino LR, Poli CHEC, Tontini JF, Corrêa GF, Somacal S, Mello RO, Leal MLR, Raimondo RFS, Riet-Correa B, Muir JP. Acacia mearnsii tannin extract and α-tocopherol supplementation in lamb diet: Effects on growth performance, serum lipid peroxidation and meat quality. *Anim. Feed Sci. Technol.* (2022) 294:115483.
40. Kafle D, Lee JH, Min BR, Kouakou B. Carcass and meat quality of goats supplemented with tannin-rich peanut skin. *J. Agric. Food Res.* (2021) 5:100159.
41. Kamel HEM, Al-Dobaib SN, Salem AZM, López S, Alaba PA. Influence of dietary supplementation with sunflower oil and quebracho tannins on growth performance and meat fatty acid profile of Awassi lambs. *Anim. Feed Sci. Technol.* (2018) 235:97–104.
42. Kamel HEM, Al-Dobaib SN, Salem AZM. Dietary supplementation of sunflower oil and quebracho tannins in sheep feeding: in vivo nutrient digestibility, nitrogen utilization and in vitro ruminal degradation kinetics. *J. Sci. Food Agric.* (2019) 99:4211–4217.
43. Karamnejad K, Sari M, Salari S, Chaji M. Effects of nitrogen source on the performance and feeding behavior of lambs fed a high concentrate diet containing pomegranate peel. *Small Rumin. Res.* (2019) 173:9–16.
44. Kazemi M. An investigation on chemical/mineral compositions, ruminal microbial fermentation, and feeding value of some leaves as alternative forages for finishing goats during the dry season. *AMB Exp.* (2021) 11:76.
45. Lee JH, Min BR. Carcass Characteristics and Meat Quality of Kiko Crossbred Male Goats as Influenced by Feeding Phytochemical Tanning Containing Supplementations. *Agric. Sci.* (2021) 12:445–463.
46. Lima PR, Apdini T, Freire AS, Santana AS, Moura LML, Nascimento JCS, Rodrigues RTS, Dijkstra J, Neto AFG, Queiroz MAÁ. Dietary supplementation with tannin and soybean oil on intake, digestibility, feeding behavior, ruminal protozoa and methane emission in sheep. *Anim. Feed Sci. Technol.* (2019) 249:10–17.

47. Mahgoub O, Kadim IT, Tageldin MH, Al-Marzooqi WS, Khalaf SQ, Ali AA. Clinical profile of sheep fed non-conventional feeds containing phenols and condensed tannins. *Small Rumin. Res.* (2008) 78:115–122.
48. Majewska MP, Kowalik B. Growth Performance, Carcass Characteristics, Fatty Acid Composition, and Blood Biochemical Parameters of Lamb Fed Diet with the Addition of Lingonberry Leaves and Oak Bark. *Eur. J. Lipid Sci. Technol.* (2020) 122:1900273.
49. Manuel-Pablo A, Elghandour MMY, Olivares-Pérez J, Rojas-Hernández S, Cipriano-Salazar M, Cruz-Lagunas B, Camacho-Diaz LM. Productive performance, rumen fermentation and carcass yield of goats supplemented with cascalote fruit (Caesalpinia coriaria J. Wild.). *Agrofor. Sys.* (2020) 94:1381–1391.
50. Mavasa NO, Ng'Ambi JW, Chitura T. Partial replacement of maize meal with high-tannin sorghum meal affects finishing and methane emissions of Pedi goats. *S. Afr. J. Anim. Sci.* (2022) 52:8–16.
51. Maxiselly Y, Chiarawipa R, Somnuk K, Hamchara P, Cherdthong A, Suntara C, Prachumchai R, Chanjula P. Digestibility, Blood Parameters, Rumen Fermentation, Hematology, and Nitrogen Balance of Goats after Receiving Supplemental Coffee Cherry Pulp as a Source of Phytochemical Nutrients. *Vet. Sci.* (2022) 9.
52. Menci R, Coppa M, Torrent A, Natalello A, Bernardo V, Giuseppe L, Alessandro P, Vincent N. Effects of two tannin extracts at different doses in interaction with a green or dry forage substrate on in vitro rumen fermentation and biohydrogenation. *Anim. Feed Sci. Technol.* (2021) 278:114977.
53. Mendo OH, Ayala Monter MA, Ortiz SL, Sánchez DH, Osorio GA, Martínez RM. Effect of Guazuma ulmifolia tannins in the diet of Pelibuey lambs on animal performance and meat characteristics. Emir. J. Food. Agric. (EJFA) (2023).
54. Min BR, Solaiman S, Gurung N, Behrends J, Eun J-S, Taha E, Rose J. Effects of pine bark supplementation on performance, rumen fermentation, and carcass characteristics of Kiko crossbred male goats. *J. Anim. Sci.* (2012) 90:3556–3567.
55. Min BR, Solaiman S, Terrill T, Ramsay A, Mueller-Harvey I. The effects of tannins-containing ground pine bark diet upon nutrient digestion, nitrogen balance, and mineral retention in meat goats. *J. Anim. Sci. Biotechnol.* (2015) 6:1–8.
56. Molina-Alcaide E, Yáñez-Ruiz DR. A comparative study of the effect of two-stage olive cake added to alfalfa on digestion and nitrogen losses in sheep and goats. *animal* (2007) 1:227–232.
57. Moreira GD, Lima P de MT, Borges BO, Primavesi O, Longo C, McManus C, Abdalla A, Louvandini H. Tropical tanniniferous legumes used as an option to mitigate sheep enteric methane emission. *Trop. Anim. Health. Prod.* (2013) 45:879–882.
58. Narjisse H, Elhonsali MA, Olsen JD. Effects of oak (Quercus ilex) tannins on digestion and nitrogen balance in sheep and goats. *Small Rumin. Res.* (1995) 18:201–206.
59. Ngámbi JW, Selapa MJ, Brown D, Manyelo TG. The effect of varying levels of purified condensed tannins on performance, blood profile, meat quality and methane emission in male Bapedi sheep fed grass hay and pellet-based diet. *Tro.p Anim. Health Prod.* (2022) 54:263.
60. Nobre PT, Munekata PES, Costa RG, Carvalho FR, Ribeiro NL, Queiroga RCRE, Sousa S, da Silva ACR, Lorenzo JM. The impact of dietary supplementation with guava (Psidium guajava L.) agroindustrial waste on growth performance and meat quality of lambs. *Meat Sci.* (2020) 164:108105.
61. Obeidat BS, Alrababah MA, Abdullah AY, Alhamad MN, Gharaibeh MA, Rababah TM, Ishmais MAA. Growth performance and carcass characteristics of Awassi lambs fed diets containing carob pods (Ceratonia siliqua L.). *Small Rumin. Res.* (2011) 96:149–154.
62. Olafadehan OA, Okunade SA, Njidda AA, Kholif AE, Kolo SG, Alagbe JO. Concentrate replacement with Daniellia oliveri foliage in goat diets. *Trop. Anim. Health Prod.* (2020) 52:227–233.

63. Olafadehan OA. Changes in haematological and biochemical diagnostic parameters of Red Sokoto goats fed tannin-rich Pterocarpus erinaceus forage diets. Vet. Arh. (2011) 81:471–483.
64. Orlandi T, Stefanello S, Mezzomo MP, Pozo CA, Kozloski G V. Impact of a tannin extract on digestibility and net flux of metabolites across splanchnic tissues of sheep. *Anim. Feed Sci. Technol.* (2020) 261:114384.
65. Osakwe II, Drochner W. Nutritive value of Morinda lucida and its fermentation parameters in West African dwarf (WAD) sheep when fed as supplement to grass hay. *Small Rumin. Res.* (2006) 64:107–115.
66. Pannell D, Kouakou B, Terrill TH, Ogunade IM, Estrada-Reyes ZM, Bryant V, Taiwo G, Idowu M, Pech-Cervantes AA. Adding dried distillers grains with solubles influences the rumen microbiome of meat goats fed lespedeza or alfalfa-based diets. *Small Rumin. Res.* (2022) 214:106747.
67. Pathak AK, Dutta N, Pattanaik AK, Chaturvedi VB, Sharma K. Effect of condensed tannins from Ficus infectoria and Psidium guajava leaf meal mixture on nutrient metabolism, methane emission and performance of lambs. *Asian-Austral. J. Anim. Sci.* (2017) 30:1702.
68. Peng K, Shirley DC, Xu Z, Huang Q, McAllister TA, Chaves A V, Acharya S, Liu C, Wang S, Wang Y. Effect of purple prairie clover (Dalea purpurea Vent.) hay and its condensed tannins on growth performance, wool growth, nutrient digestibility, blood metabolites and ruminal fermentation in lambs fed total mixed rations. *Anim. Feed Sci. Technol.* (2016) 222:100–110.
69. Pérez BIC, Rojas-Román LA, Estrada-Angulo A, Muro OC, Barreras A, Plascencia A. Effects of long-term supplementation of different levels of tannins extract on meat quality and carcass traits of hairy lambs. Adv. Anim. Vet. Sci. (2021) 9:1973–1977.
70. Pimentel PRS, Pellegrini CB, Lanna DPD, Brant LMS, Ribeiro C, Silva TM, Barbosa AM, da Silva Júnior JM, Bezerra LR, Oliveira RL. Effects of Acacia mearnsii extract as a condensed-tannin source on animal performance, carcass yield and meat quality in goats. *Anim. Feed Sci. Technol.* (2021) 271:114733.
71. Priolo A, Waghorn GC, Lanza M, Biondi L, Pennisi P. Polyethylene glycol as a means for reducing the impact of condensed tannins in carob pulp: effects on lamb growth performance and meat quality. *J. Anim. Sci.* (2000) 78:810–816.
72. Priolo A, Bella M, Lanza M, Galofaro V, Biondi L, Barbagallo D, Salem H Ben, Pennisi P. Carcass and meat quality of lambs fed fresh sulla (Hedysarum coronarium L.) with or without polyethylene glycol or concentrate. *Small Rumin. Res.* (2005) 59:281–288.
73. Priolo A, Vasta V, Fasone V, Lanza CM, Scerra M, Biondi L, Bella M, Whittington FM. Meat odour and flavour and indoles concentration in ruminal fluid and adipose tissue of lambs fed green herbage or concentrates with or without tannins. *Animal* (2009) 3:454–460.
74. Raju J, Sahoo B, Chandrakar A, Garg AK, Mohanta RK. Effect of varied sources of tannin on micro-mineral bioavailability in goats fed oak leaves based diets. *Anim. Nutr. Feed Technol.* (2018) 18:25–35.
75. Reynolds D, Min BR, Gurung N, McElhenney W, Lee JH, Solaiman S, Bolden-Tiller O. Influence of tannin-rich pine bark supplementation in the grain mixes for meat goats: Growth performance, blood metabolites, and carcass characteristics. *Anim. Nutr.* (2020) 6:85–91.
76. Rojas-Román LA, Castro-Pérez BI, Estrada-Angulo A, Angulo-Montoya C, Yocupicio-Rocha JA, López-Soto MA, Barreras A, Zinn RA, Plascencia A. Influence of long-term supplementation of tannins on growth performance, dietary net energy and carcass characteristics: Finishing lambs. *Small Rumin. Res.* (2017) 153:137–141.
77. Salami SA, Valenti B, O'Grady MN, Kerry JP, Mattioli S, Licitra G, Luciano G, Priolo A. Influence of dietary cardoon meal on growth performance and selected meat quality parameters of lambs, and the antioxidant potential of cardoon extract in ovine muscle homogenates. *Meat Sci.* (2019) 153:126–134

78. Sánchez N, Mendoza GD, Martínez JA, Hernández PA, Camacho Diaz LM, Lee-Rangel HA, Vazquez A, Flores Ramirez R. Effect of Caesalpinia coriaria fruits and soybean oil on finishing lamb performance and meat characteristics. *Biomed. Res. Int.* (2018) 2018:9486258.
79. Santos SK dos, Rosset M, Miqueletto MM, Jesus RMM de, Sotomaior CS, Macedo REF de. Effects of dietary supplementation with quebracho tannins on oxidation parameters and shelf life of lamb meat. *Food Sci. and Technol.* (2021) 42:e55920.
80. Sena JAB, Villela SDJ, Santos RA, Pereira IG, Castro GHF, Mourthé MHF, Bonfá CS, Martins PGMA. Intake, digestibility, performance, and carcass traits of rams provided with dehydrated passion fruit (Passiflora edulis f. flavicarpa) peel, as a substitute of Tifton 85 (Cynodon spp.). *Small Rumin. Res.* (2015) 129:18–24.
81. Seyedin SMV, Mojtahedi M, Farhangfar SH, Ghavipanje N. Partial substitution of alfalfa hay by Berberis vulgaris leaf modulated the growth performance, meat quality and antioxidant status of fattening lambs. *Vet. Med. Sci.* (2022) 8:2605–2615.
82. Śliwiński BJ, Kreuzer M, Wettstein H-R, Machmüller A. Rumen fermentation and nitrogen balance of lambs fed diets containing plant extracts rich in tannins and saponins, and associated emissions of nitrogen and methane. *Arch. Anim. Nutr.* (2002) 56:379–392.
83. Sinz S, Leparmarai PT, Liesegang A, Ortmann S, Kreuzer M, Marquardt S. Effects of dietary grapeseed extract on performance, energy and nitrogen balance as well as methane and nitrogen losses of lambs and goat kids. *Br. J. of Nutr.* (2021) 125:26–37.
84. Solaiman S, Thomas J, Dupre Y, Min BR, Gurung N, Terrill TH, Haenlein GFW. Effect of feeding sericea lespedeza (Lespedeza cuneata) on growth performance, blood metabolites, and carcass characteristics of Kiko crossbred male kids. *Small Rumin. Res.* (2010) 93:149–156.
85. Soltan YA, Morsy AS, Sallam SMA, Lucas RC, Louvandini H, Kreuzer M, Abdalla AL. Contribution of condensed tannins and mimosine to the methane mitigation caused by feeding Leucaena leucocephala. *Arch. Anim. Nutr.* (2013) 67:169–184.
86. Soltani Nezhad B, Dayani O, Tahmasbi R, Khezri A. Effects of Replacing Alfalfa Hay and Wheat Straw by Pistachio by-Product Silage and Date Waste on the Performance and Blood Parameters of Fattening Lambs. *Iran. J. Appl. Anim. Sci.* (2016) 6:587–593.
87. Taethaisong N, Paengkoum S, Kaewwongsa W, Onjai-Uea N, Thongpea S, Paengkoum P. The Effect of Neem Leaf Supplementation on Growth Performance, Rumen Fermentation, and Ruminal Microbial Population in Goats. *Animals* (2023) 13:890.
88. Uushona T, Chikwanha OC, Katiyatiya CLF, Strydom PE, Mapiye C. Production and meat quality attributes of lambs fed varying levels of Acacia mearnsii leaf-meal as replacement for Triticum aestivum bran. *Meat Sci.* (2023) 196:109042.
89. Vasta V, Pennisi P, Lanza M, Barbagallo D, Bella M, Priolo A. Intramuscular fatty acid composition of lambs given a tanniniferous diet with or without polyethylene glycol supplementation. *Meat Sci.* (2007) 76:739–745.
90. Vasta V, Priolo A, Scerra M, Hallett KG, Wood JD, Doran O. Δ9 desaturase protein expression and fatty acid composition of longissimus dorsi muscle in lambs fed green herbage or concentrate with or without added tannins. *Meat Sci.* (2009) 82:357–364.
91. Vasta V, Mele M, Serra A, Scerra M, Luciano G, Lanza M, Priolo A. Metabolic fate of fatty acids involved in ruminal biohydrogenation in sheep fed concentrate or herbage with or without tannins. *J. Anim. Sci.* (2009) 87:2674–2684.

92. Wang S, Terranova M, Kreuzer M, Marquardt S, Eggerschwiler L, Schwarm A. Supplementation of pelleted hazel (Corylus avellana) leaves decreases methane and urinary nitrogen emissions by sheep at unchanged forage intake. *Sci. Rep.* (2018) 8:5427.
93. Wang Z, Guo L, Ding X, Li F, Xu H, Li S, Wang X, Li K, Yue X. Supplementation of chestnut tannins in diets can improve meat quality and antioxidative capability in Hu lambs. *Meat Sci*. (2023) 206:109342.
94. Wu P, Fu X, Wang H, Hou M, Shang Z. Effect of silage diet (sweet sorghum vs. whole-crop corn) and breed on growth performance, carcass traits, and meat quality of lambs. *Animals* (2021) 11:3120.
95. Yisehak K, Kibreab Y, Taye T, Ribeiro Alves Lourenço M, Janssens GPJ. Response to dietary tannin challenges in view of the browser/grazer dichotomy in an Ethiopian setting: Bonga sheep versus Kaffa goats. *Trop. Anim. Health Prod.* (2016) 48:125–131.
96. Yusuf AL, Adeyemi KD, Roselina K, Alimon AR, Goh YM, Samsudin AA, Sazili AQ. Dietary supplementation of different parts of Andrographis paniculata affects the fatty acids, lipid oxidation, microbiota, and quality attributes of longissimus muscle in goats. *Food Res. Int.* (2018) 111:699–707.
97. Zhao MD, Di LF, Tang ZY, Jiang W, Li CY. Effect of tannins and cellulase on growth performance, nutrients digestibility, blood profiles, intestinal morphology and carcass characteristics in Hu sheep. *Asian-Australas J. Anim. Sci*. (2019) 32:1540.
